# High intra-specific variation in avian body condition responses to climate limits generalisation across species

**DOI:** 10.1371/journal.pone.0192401

**Published:** 2018-02-21

**Authors:** Nina McLean, Henk P. van der Jeugd, Martijn van de Pol

**Affiliations:** 1 Division of Evolution, Ecology & Genetics, Research School of Biology, The Australian National University, Canberra, Australia; 2 Department of Animal Ecology, Netherlands Institute of Ecology (NIOO-KNAW), Wageningen, The Netherlands; 3 Vogeltrekstation - Dutch Centre for Avian Migration and Demography, Netherlands Institute of Ecology (NIOO-KNAW), Wageningen, The Netherlands; Hungarian Academy of Sciences, HUNGARY

## Abstract

It is generally assumed that populations of a species will have similar responses to climate change, and thereby that a single value of sensitivity will reflect species-specific responses. However, this assumption is rarely systematically tested. High intraspecific variation will have consequences for identifying species- or population-level traits that can predict differences in sensitivity, which in turn can affect the reliability of projections of future climate change impacts. We investigate avian body condition responses to changes in six climatic variables and how consistent and generalisable these responses are both across and within species, using 21 years of data from 46 common passerines across 80 Dutch sites. We show that body condition decreases with warmer spring/early summer temperatures and increases with higher humidity, but other climate variables do not show consistent trends across species. In the future, body condition is projected to decrease by 2050, mainly driven by temperature effects. Strikingly, populations of the same species generally responded just as differently as populations of different species implying that a single species signal is not meaningful. Consequently, species-level traits did not explain interspecific differences in sensitivities, rather population-level traits were more important. The absence of a clear species signal in body condition responses implies that generalisation and identifying species for conservation prioritisation is problematic, which sharply contrasts conclusions of previous studies on the climate sensitivity of phenology.

## Introduction

A major aim of climate change ecology is to understand why species differ in their sensitivity to climate change and identify which species are most at risk [1]. To answer these questions, studies typically assume—sometimes explicitly, often implicitly—that climate responses from individual studies are representative for the species as a whole. For example, comparative meta-analyses often use values reported in local population studies to investigate interspecific variation in climate responses (e.g. [1–3]), and the IUCN Red List uses evidence from studies on specific population(s) to argue that the species as a whole might be under threat from climate change [4]. Empirical studies on the effects of climate change rarely consider variability in responses among populations of the same species [5–7]. Typically a single value is used which is thought to adequately reflect species-specific responses to changing environments, essentially assuming that within-species variation is negligible [8–11].

However, is the concept of a species’ climate response useful for generalisation and prediction? If responses to climate change vary substantially within species—which they often appear to do [12–19]—then generalising the response to a single species signal may not be accurate, nor meaningful. For instance, a single species signal would be unrepresentative if populations showed different climate-driven responses at their low-latitude range margin compared to their poleward range margins [11,20,21]. In such a situation it is important to properly account for this intra-specific variation when modelling environmental responses, as it can help us to understand the mechanisms underlying responses to environmental change, and to better predict their effects [8,10]. For instance, intraspecific variation can reduce the overall effects of climate change on the species because some populations may be less affected, essentially buffering the overall impacts [6,22,23]. This phenomenon is known as the portfolio effect, where spatial or genotypic diversity can dampen variation in total population abundance [24].

Identifying life-history and ecological traits that can explain and predict differences in sensitivities is currently of particular importance for making effective conservation and management decisions [1,25]. For most species, we lack the sufficient data to make reliable direct estimates of their climate sensitivities, and consequently nowadays conservation organisations often indirectly predict the climate sensitivities of data-deficient species based solely on their species traits [25]. However, when trying to make generalisations across species, few comparative studies consider variation among populations within species. When there is high intraspecific variation, species life-history traits (e.g. life-expectancy), which are the main focus of most comparative studies, are likely to be of little predictive power. Instead, if a species is not responding consistently across different populations, it suggests that local, population-specific traits (e.g. habitat type) could be more important.

In order to provide some yard-stick for what ‘a lot’ of intraspecific variation might be, and therefore when local rather than species traits might be more important, comparative analyses that include both intra- and inter-specific variation are needed. In such studies, it is possible to determine whether populations of the same species are more alike than populations of different species, suggesting a species signal exists [6,8]. Ample comparative studies exist that compare interspecific variation and quite some studies compare intraspecific variation of one or a few species (e.g. [12,13,16–18]). However, apparently there are so few species with sufficient intraspecific information [26] that intraspecific analyses of many species has seldom been achieved.

As far as we are aware, only three studies have systematically investigated the amount of within- to among-species variation in responses to climate change. Rubolini et al. [5] and Thackeray et al. [7] both found strong species-signals, with about 50% of the variance in observed changes in phenology attributed to differences among species (although the latter study did not explicitly interpret this result in the context of species signals). In contrast, Malyshev et al. [6] found that only roughly 10% of the variance was due to among-species variation in plant growth responses to drought and winter frost. Thus, the little evidence available in the literature suggests a strong species signal for phenological changes, but we have no indication as to whether this might hold true more generally for other types of climate responses, particularly for key state variables (body mass, growth) and vital rates (reproduction, survival) that determine the population responses relevant for conservation.

Body condition (mass corrected for size; i.e. amount of fat and protein reserves; [27]) is a key state variable that affects vital rates [28–31] and thereby is likely important for population dynamics (e.g. [32]). It is now becoming clear that body condition and mass of avian species around the world are changing substantially over time and with climate [28,29,31–39]. However, despite being identified as one of the three major responses to climate change, the impacts of climate on body condition (and body size) have been less well studied than responses in phenology and range shifts [40]. Of the few studies that investigate the relationship between temperature and body condition directly, warmer temperatures have often been found to result in decreased juvenile and adult body mass [28–30,34,41]. This can be either through indirect effects, as temperature can alter the amount of food resources available [30], or through direct effects on energetics [29,34,42]. However, we still do not have a good understanding of the mechanisms underlying environmentally-driven changes in body condition [43].

Despite the importance of body condition, our understanding of climatic effects on body condition is limited. The literature has mostly focused on temperature responses, but other climatic variables could also have important influences on body condition (see Box 1 for a description of other climate variables and potential underlying mechanisms). We also have little understanding of how the effect of climate on body condition varies among different populations. This is in part because the majority of research investigates changes over time, which makes general patterns difficult to interpret as the direction of changes will depend on the climate in the local region [43]. What’s more, the time periods (e.g. winter, spring) during which climate has the strongest impact on body condition are rarely systematically tested and so are still poorly understood [43–45]. Typically only linear responses have been considered, so it is unclear as to whether body condition responses might be non-linear (but see [38,39]).

**Box 1. Potential effects of various climate variables on body condition.**

A range of climate variables could impact body conditions of birds through both direct and indirect means. The bulk of the literature that investigates body condition responses to climate focuses on temperature, and to a lesser extent rainfall. Temperature could have direct effects on condition via a number of mechanisms. Temperature has a direct effect on their energetics [42]. Warmer temperatures can result in overheating or decreased foraging efficiency [28,76]. Birds might strategically down-regulate their body mass under warmer conditions (as they may not need as much body fat for warmth) which could reduce time spent feeding and improve flight performance [77,78]. Changes in rainfall can impact freshwater supplies, which can have consequences for hydration [28]. Changes in temperature and rainfall may have indirect effects on body mass through changes in food availability, perhaps through mismatches in peak food abundances [30,79] or by exacerbating parasites and diseases which impact on the health of birds [28].

However, other climatic variables could arguably also impact body condition. For instance, humidity can impact heat retention and fuel composition which can, in turn, impact lean mass [76,80]. Birds rely primarily on evaporative cooling from cutaneous and respiratory surfaces for heat dissipation, which is much less effective in high humidity [76]. Wind speed can have multiple effects on birds that may impact their body condition. Wind speed affects bird energetics [81,82], body temperature [83], field metabolic rate [81,82], it reduces thermal resistance of the feathers such that they change their orientation [82,84], and it affects the movement of migratory land birds. Strong wind has been found to have a negative impact on the body condition of chicks [81,82]. Alternatively, high wind speeds may allow some birds to fly faster and reach foraging sites more easily, resulting in increased body condition [85].

The amount of sunshine (i.e. cloudiness) may impact foraging behaviour, movement and body temperature. Exposure to bright sunlight might make birds easier to detect by predators, and visual glare could reduce their ability to monitor the environment effectively [82]. In cold environments, heat gain from solar radiation can reduce the costs of foraging [82]. However, Konarzewski & Taylor [81] found that sunshine did not impact chick mass in Little Auks, nor were feeding rates affected by cloud cover in Guillemots [86]. The consequences of changes in daily temperature range in endotherms are mostly unknown, but are most likely associated with increased thermal stress [87]. Increased temperature range can impact mortality, egg size and cost of energy expenditure [87,88].

As a result of this missing knowledge, there are currently no future projections for how body condition is likely to be impacted under future climate scenarios. Projecting the ecological responses of future climate changes using recent observed effects is now a major challenge in climate ecology [46]. As the climate is predicted to continue to change in the future, conservation plans and action will rely on our ability to accurately project impacts [47,48].

Here we use an extensive 21-year data set from 80 Dutch constant effort sites to investigate (1) how avian body condition responds to changes in climate, and (2) how consistent these responses are both across and within 46 common passerine species. We also project how species and populations are likely to change under future climate scenarios. We first test how sensitive species and populations are to six climate variables that we hypothesise as having important effects on body condition (temperature, rainfall, humidity, sunshine, daily temperature range (DTR) and wind speed; see Box 1), and determine the time period (testing all options over a full year) in which the effect is most marked. Subsequently, we integrate the sensitivities of populations and projected changes in climate to predict body condition responses to future climatic conditions in 2050. Second, we ask whether there is a species signal, or instead if populations of the same species respond as differently as populations of different species, by comparing the relative amounts of within- to among-species variation. We investigate whether species and population traits can explain variability in responses among species and populations. We predict that if intra-specific variation is high, species traits (e.g. life-expectancy) and phylogenetic distance (a proxy for unknown species traits) will be of less importance for predicting body condition responses to climate; rather population traits (e.g. habitat type) and geographic distance (closer sites are more similar) should be more appropriate.

## Materials and methods

### Body condition data for common Dutch bird species

Approval of the work by an ethics committee is not required for catching and banding birds in the Netherlands. Under Dutch law, catching and banding birds requires a banding license which each of the banders have obtained from the bird banding scheme. The Dutch constant effort site project has run over a period of 21 years at 80 field sites across the Netherlands ([49]; 1994–2014; see Figure A in S1 Appendix for map). The project followed a standardised protocol [50] where mist netting is carried out with a constant effort from the 12^th^ April until the 14^th^ August, 12 times per year. We focussed on 46 common passerine species of which in total 174,875 birds were caught. Not all sites collected observations over the 21 years of the study period (mean = 10 years, range = 1–21 years); 10 species were captured in less than 7 different sites, and for this reason excluded from any intra-specific analyses. Captured birds are ringed and morphometric measurements taken, including body mass (grams), wing length (maximum chord measurement; [51]), sex and age-class (typically juvenile or adult; based on the plumage of the bird). We estimated body mass corrected for size by taking the residuals from the linear regression of body mass on wing length; this means that our measure of body condition is more of a measure of body fat [27,29,31]. When investigating the effects of climate on body size or condition, wing length is generally thought to be the best single linear predictor of structural size for passerines [29,52]. Although wing length has been found to be affected by climate [for example 53], we found no change in wing length over the length of the study, suggesting that our measure of body condition was not affected by any changes in wing length.

### Climatic data

Our knowledge of which climate variables are important for body condition is limited, as previous studies have mostly focused on the effects of temperature or, less frequently, rainfall (for example [28,29,33,34,37]). We suggest a number of plausible weather signal hypotheses (see Box 1). Consequently, we look at the effects of six climatic variables: daily windspeed (in 0.1 m/s), mean daily temperature (over 24 hours, °C), mean relative humidity (percent), longest possible daily sunshine duration (percent), daily sum precipitation (in 0.1 mm), and daily temperature range (DTR, difference in minimum and maximum daily temperatures in °C). Daily records of each of the six variables over the study period were available from the Royal Netherlands Meteorological Institute (KNMI) for 37 weather stations across the Netherlands. The biological data from each site was matched with the closest weather station (mean distance 17 km; see Table B in S1 Appendix).

Climate projections were available for all climate variables except sunshine, based on a regional climate model from the Royal Netherlands Meteorological Institute (KNMI). We chose to use the most extreme of the four available climate scenarios (‘WH’), as the best case scenario is thought unlikely [54], but also because using a worst case scenario can be more useful for conservation decisions. The WH scenario assumes a high global temperature change (around 2°C by 2050) and strong changes in air stream patterns in the Netherlands [55]. Under this scenario, temperature and wind speeds are projected to increase across all seasons, while humidity is expected to decrease. Daily temperature ranges are projected to decrease in all seasons except summer where it increases. Conversely, rainfall is projected to increase in all seasons except summer where it decreases. We assumed that there would be little geographic variation in projected exposure across the small spatial scale of the Netherlands and therefore use the same climate exposure for all populations (i.e. the furthest sites are only around 250km apart, see Figures A & B in S1 Appendix; [47]). For all climate variables except wind (for which only one annual estimate was available), we matched the projected exposure for each season (winter/ spring/ summer/ autumn) to the season when the climate window occurred (see later). In cases where the climate window spanned more than one season, we averaged those seasons’ projected exposures.

### Species and site trait data

We identified a number of hypotheses about species and population traits that could potentially explain differences in body condition responses to climate. We predict that if intra-specific variation is high, population-level traits will likely explain most variation. However, species-level traits may still be important for explaining some variation. We tested four species level (body size, migratory strategy, habitat preference and life-expectancy) and two site level hypotheses (habitat and population density), see Box 2 for rationale.

**Box 2. Species and site level hypotheses to explain intra- and inter-specific variation in climate responses.**

**Species level hypotheses**
1. Body mass responds differently to changes in climate in larger and smaller bird species. Body size affects a range of biological processes including water requirements, thermoregulation, energy and mass acquisition and utilisation rates [40]. Size plays an important role with climate, as smaller individuals are generally found at lower latitudes where climates are warmer (Bergmann’s rule; [40, 89]).
2. Body mass responses differ among species with different migratory strategies (resident, short- and long-distant migrants). Environmental conditions can have stronger effects on migrants because they have a higher chance of mismatches with important resources [90–93]. Migratory species are also influenced by conditions in other areas [94]. Additionally, migrant species may be less likely to increase their body mass as increased fat can have strong negative effects during migrations [95].
3. A species’ preferred habitat type could alter their physiological response to climate. Food availability might change if forest phenology is advanced, affecting invertebrate timing and availability [72,90,92]. Marshes or reeds are more stable because they grow throughout spring and summer [72]. Also, some habitat types might interact with climate by providing more refugia [90]. Forests can buffer extreme weather (i.e. windy conditions, shade), whereas marshland areas provide standing water but can fluctuate strongly with precipitation and temperature [90].
4. Species with different longevity may adopt different trade-offs when dealing with climatic impacts on body mass because life-expectancy can alter strategies under poor environmental conditions [96]. For instance, shorter lived species may require a growth rate out of proportion to food availability, as species that are under time pressure to attain a certain size at a given time will often sacrifice future fitness by growing out of proportion to resource availability [97]. Long-lived species might be more flexible as they do not need to put on the weight as quickly.

**Site level hypotheses**
1. Body mass responses may differ between habitat types (wet or dry) as changes in climate can affect freshwater supplies, making it difficult for birds to hydrate [28].
2. Population density could act as a proxy for site quality, where populations in better quality sites may be more resilient to changes in climate [28].

Furthermore, in the absence of other *a priori* hypotheses about explanatory traits, phylogenetic (or geographic) distances among species may be able to act as a proxy for predicting species’ (or site) responses [56]. Phenotypic differences between species and populations are expected to rise over eco-evolutionary time, such that closely related species and proximate populations should respond more similarly to environmental change [1,57]. Similarly, geographic distance among sites could explain site-variation in body condition responses because closer populations are expected to have more similar environments, and thus geographical distance could be acting as a proxy for some unknown environmental variable [58]. We therefore examined whether phylogenetic (i.e. time of divergence in millions of years) and spatial (Euclidian in km) distance could predict the amount of dissimilarity in climate responses among species and sites.

Body size estimates were calculated as the mean body mass across all individuals for each species. Species’ preferred habitat type was assigned into the categories urban (garden), woodland, wet (reed bed, wet scrub) and open (dry scrub) based on [59]. Life-span was adult life-expectancy, calculated as 1/adult annual survival. Population density was calculated as the number of individuals of the same species caught (per meter of net per day) for each site averaged across all years. Population habitat type needed to be grouped into wet (reed bed, wet scrub) or dry (dry scrub, garden, woodland) because some habitats were rare. To determine phylogenetic distance, we downloaded 1,000 different possible phylogenetic trees from a pseudoposterior distribution from birdtree.org [57,60] (Figure G in S1 Appendix).

### Statistical analyses

We explore avian body condition responses to changes in climate using species and site-specific sensitivity values (how strongly body condition is affected by a change in specific climate variables) and future projections (projected change in percent body condition by 2050 due to all climate variables combined) based on regional climate model projections [55].

#### Climate variables and windows

**Fig 1:**
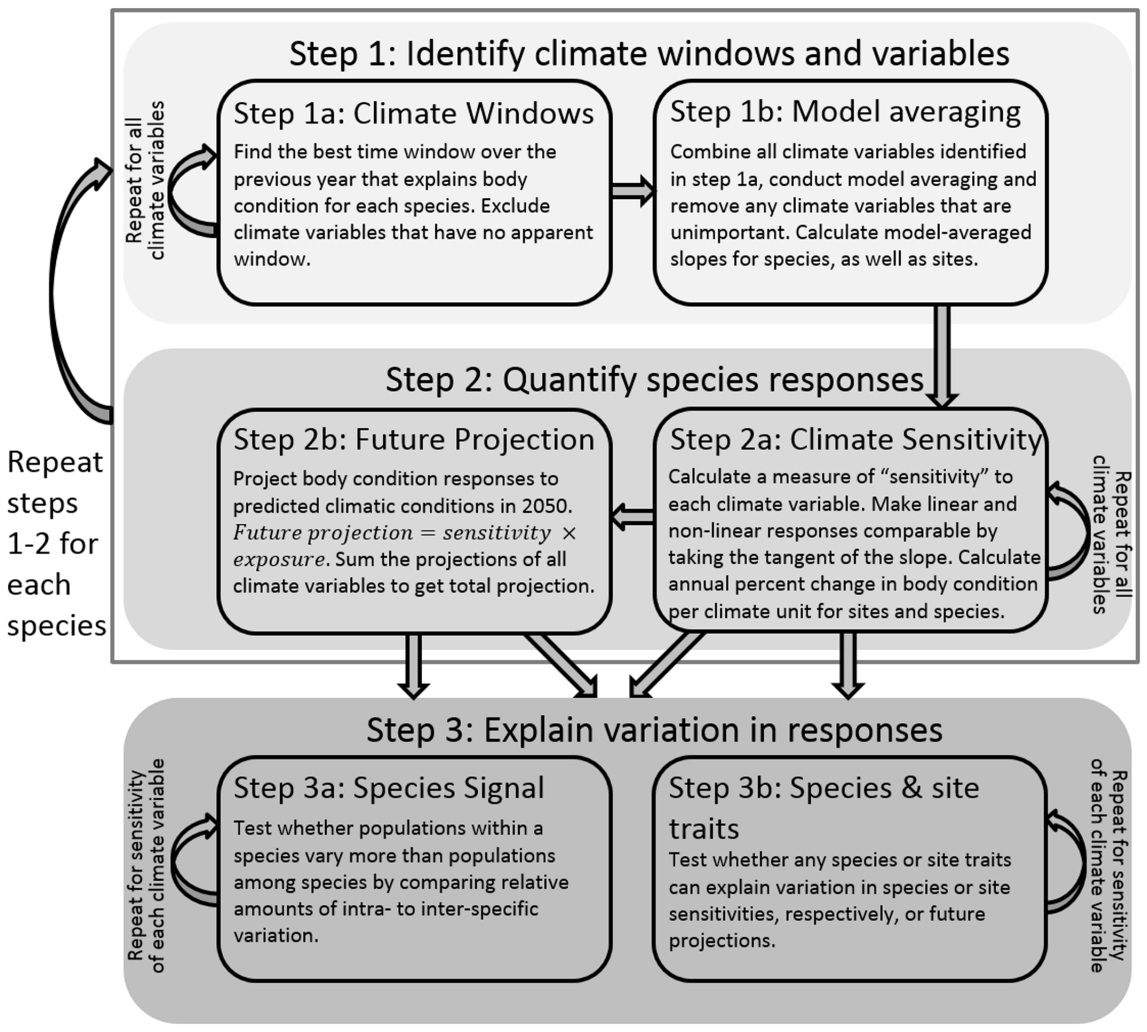
Conceptual diagram summarising the three main steps of the analyses. **(1)** identifying the climate variables that affect each species and over which time periods (i.e. climate windows), (2) quantifying species and population responses to climate, and (3) investigate inter and intraspecific variation in climate responses. Step 3a investigates the relative amounts of intra- and inter-specific variation to ask how consistent responses are and whether there is a species signal. While in step 3b, comparative analysis is used to test for any species or site traits (e.g. phylogenetic relatedness or habitat type) that explain differences among species or sites sensitivities and future projections. Steps 1 and 2 are carried out on each species individually, while in Step 3 all species are combined.

The first step was to identify which climate variables impacted body condition, and over which periods. For each species and climate variable we performed a climate window analysis to identify the time period during which the variable explained the most variation in body condition, using the R package *climwin* (Fig 1 Step 2a; [61]). This allowed us to take an exploratory approach, as it considers all possible combinations of consecutive days for the whole year (i.e. the 365 days before the end of the sampling season on the 15^th^ August) to identify the ‘best’ possible window (see S1 Appendix for details). As such, all time periods over all seasons (i.e. summer or winter etc.) were investigated. Randomisation techniques were used to assess the likelihood that the best time window is actually a spurious result of overfitting ([44]; see S1 Appendix). We added the following predictor variables to these models (in addition to mean climate) to account for the confounding effects of age (juvenile or adult), sex (if identifiable for that species), day within the season, time of capture, and the random effect ‘Individual ID’ (some individuals were caught repeatedly). We investigated both linear and quadratic relationships between climate and body condition because we had no *a priori* reason to suspect the relationships would be linear.

As the *climwin* analysis tests only a single climate variable at a time, we combined the best windows of each climate variable into one model in order to account for the effects of multiple climate variables. We next included all climatic variables (grand-mean centred; and linear or quadratic) that were found to have a climate signal plus the baseline variables (age, sex, day within season, time, individual ID) into a single model explaining variation in body condition. For example, if a species was affected by all six climate variables, the full model would be:

$$Body\;Mass=Temperature+Rain+Wind+DTR+Humidity+Sunshine+Age+{Time+Time}^{2}+{Season+Season}^{2}+Sex+(1|Individual\;ID)$$

Model selection and averaging was used to determine which climate variables were important and to calculate parameter estimates after accounting for the other climate variables [62]. For species that were found to be affected by multiple climate variables, we compared models with every possible combination of those climate variables, while including the climate-unrelated explanatory variables (age class, observed sex, time, season and individual ID) in all models. We subsequently excluded climate variables if they were not present in any models within 2 delta AICc units of the best model [62], as this would suggest that their effects were of low biological relevance, or if they appeared to be uninformative (“hitchhiker”) variables (S1 Appendix for definition; [63]). To calculate the model-averaged slope estimates for each site, an interaction term between the site and the climate variable was included.

Collinearity among climate variables (defined as r>0.6) was rare; collinear climate variables were always kept in the same models when testing the different combinations of variables (i.e. both always included or excluded from the same models). In this way, the effects of the two correlated climatic variables were always measured together, which yields unbiased parameter estimates ([64]; see S1 Appendix for details). We checked whether sensitivities to climate variables differed substantially between juveniles and adults, and between males and females, however there was no strong difference between groups, thus we only present results for adult females (see S1 Appendix for details). All models were fitted using the *lmer* function of the *lme4* package in the R statistical package [65] and the *MuMIn* package was used for model averaging and selection [66].

#### Climate sensitivities

In order to compare linear and non-linear responses, we calculated a measure of “sensitivity” to each climate variable (Fig 1 Step 2a). Our measure of sensitivity was the tangent at the mean climate (i.e. the first derivative of the climate regression function or the local slope estimate at the mean value of the climate variable of interest; Figure F in S1 Appendix; [67]). This gives the change in body condition (grams) per climate unit at the mean climate, or the climate sensitivity in average climatic conditions. By taking the slope at the mean climate we can investigate projections for the near future. The mean climate was calculated across all sites and years. We excluded any sites that were measured solely in years in which the climatic conditions were above or below the mean climatic conditions over the entire study period, as we did not want to extrapolate beyond the available data (10 sites on average across all species and climate variables, ranging from 0–58 sites). In order to compare climate sensitivities among species that differed in body size (i.e. a change of 1 gram would be quite different for larger or smaller birds), we used the percent change in body condition per climate unit.

#### Future projections

We projected the change in percent body condition (B) by 2050 by multiplying each species’ sensitivity ($\frac{\partial B}{\partial C_{i}}$; change in condition per climate unit) with their projected exposure based on climate scenarios for 2050 ($\frac{dC_{i}}{dT}$; predicted change in climate over time). The sum across all climate variables gives the overall future projection estimate ($\frac{dB}{dT}$; change in condition over time due to the combined effect of all climate variables) (Eq 1).

$$\frac{dB}{dT}=\sum_{i}\left(\frac{\partial B}{\partial C_{i}}*\frac{dC_{i}}{dT}\right)$$ (1)

where B represents body condition; C_i_ the climate variable *i*, and T time. We calculated future projections to climate change for each site. For a single climate variable, their future projection could be small for several reasons. A species could be highly sensitive, but have only a small projected change in climate by 2050 (i.e. little exposure). Similarly, high exposure with low sensitivity will result in small future changes. When this occurs, the climate variable will have a low contribution to the overall future projection for the population or species.

#### Species signal

**Fig 2:**
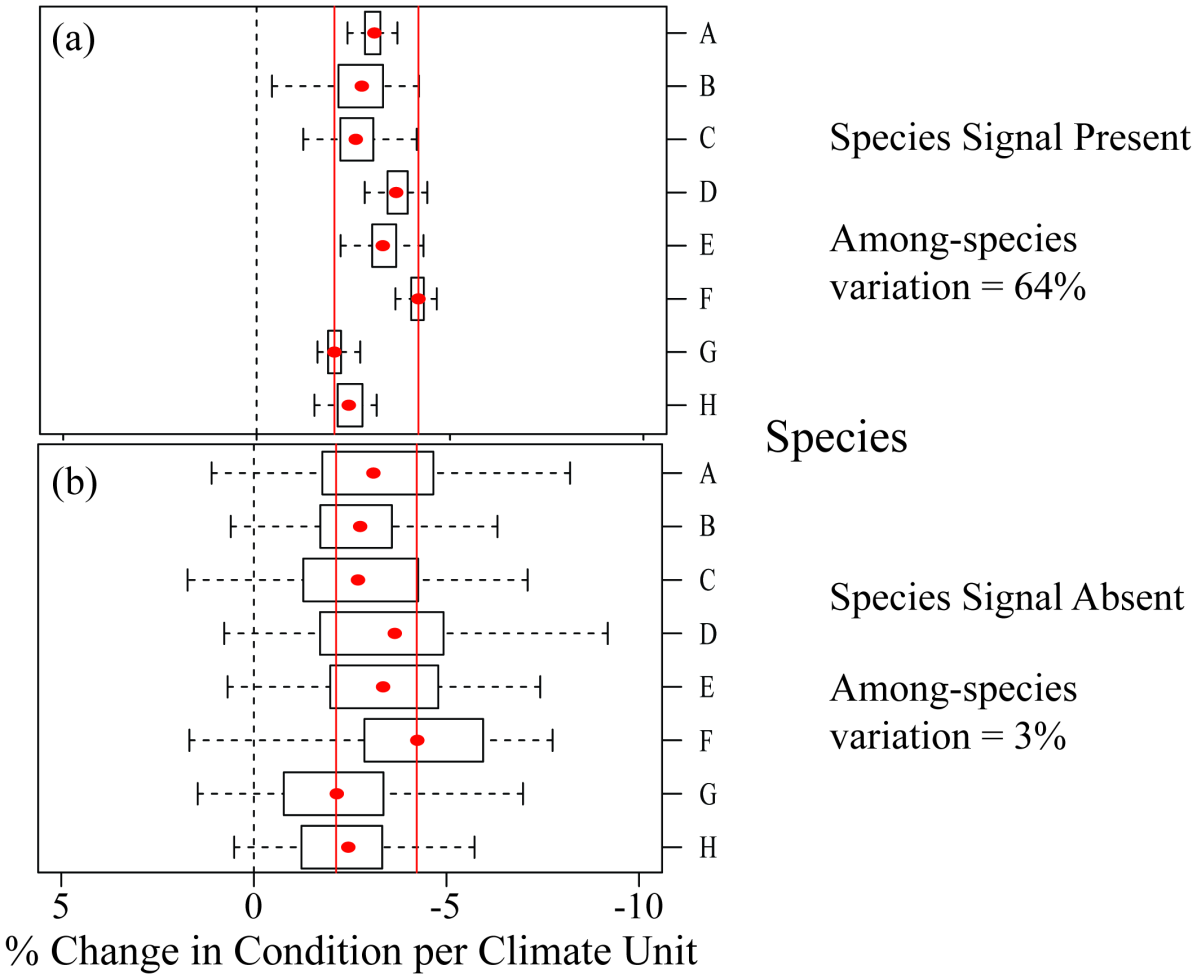
Illustration of hypothetical intra-specific and inter-specific variation in sensitivities to climate. Plot (a) shows an example where a species signal is present. Here, the correlation between the climate sensitivity of two populations of the same species is much higher than the correlation between two populations of different species. The percentage of among-species variation explained is 64%, which suggests that population variation is low compared to among-species variation. Plot (b) shows an example where a species signal is absent. Here, the correlation between the climate sensitivity of two populations of the same species is lower than the correlation between two populations of different species (i.e. population sensitivities within a single species vary just as much as much as population sensitivities among different species). As such, the percentage of among-species variation explained is a much smaller 3%. The sensitivity estimates for each species is shown by the red points, while the black boxplots show the distribution of population sensitivity estimates (intraspecific or among-site variation in climate sensitivity). The two red vertical lines show the minimum and maximum of the species’ sensitivity estimates (i.e. the range of the red points).

To determine whether a species signal exists, we quantified the amount of among- and within-species variation in sensitivity and future projections. We ran an intercept-only mixed ‘variance component model’ model [68] with site-specific sensitivity estimates as the response variable (weighted by the inverse standard error of the sensitivities), and ‘species’ as a random intercept term. This was performed for each climate variable separately. To estimate the relative amount of variation in body condition response that was due to species differences, we compared the ratio of the among-species variation (estimate of the variance of the random effect ‘species’) to the total variation (the sum of the ‘species’ and residual ‘population’ variance estimate) [8,11]. This ratio can also be interpreted as the intra-class correlation coefficient (i.e. the similarity between the climate responses of populations of the same species; [68]). A value close to zero suggests that among-species variation is low, while population variation is high, indicating that a species signal might not be present (Fig 2). Alternatively, a value close to one suggests that the correlation between the climate sensitivity of two populations of the same species was much higher than the correlation between two populations of different species [8].

This method unfortunately introduces sampling variance into the residual ‘population’ variance estimate because it is carried out in two steps (uses model-based climate sensitivity estimates with varying levels of error as the dependent variable in a second model). However, this was unavoidable given that this analysis could not be carried out on the raw body condition response data due to the different climate variables, and also included linear and non-linear responses for each species. We took two steps to address this issue. First, each observed climate response was weighted by its uncertainty (1/standard error of response estimate) to reduce the inflating effect of sampling variance on the residual (population) variance. Additionally, we investigated the impact of sampling variance on response estimates by assessing whether a relationship existed between the number of years a site was sampled and how different (absolute deviation) each population was from the species mean. We conducted a quadratic regression on the absolute residuals over the number of years sampled and found no relationship between them (within 0.04 AICc units of the null model), suggesting that inflating effects of sampling variance on variance components were negligible.

#### Species and site traits

To investigate whether species and population traits can explain variability in responses, we ran a mixed model for each hypothesis (Box 2), with either sensitivities or overall future projections as the response variables, trait as the explanatory variable, and species as the random intercept term. All models were weighted by the inverse standard error of the sensitivities. We compared the AICc value of each to a null (intercept only) model. Pairwise phylogenetic and geographic distance were related to dissimilarity in climate sensitivity (the absolute difference in sensitivities between two species or sites), and phylogenetic distance was also related to whether species were impacted by the same climate variables (i.e. whether both species are affected by that climate variable, or if one species is affected by that climate variable, but the other is not) (see S1 Appendix for details). If the relationship between phylogenetic (or geographic) distance and dissimilarity is positive, more closely related (spatially proximate) species respond more similarly to climate variables.

## Results

### Climate sensitivities

**Fig 3:**
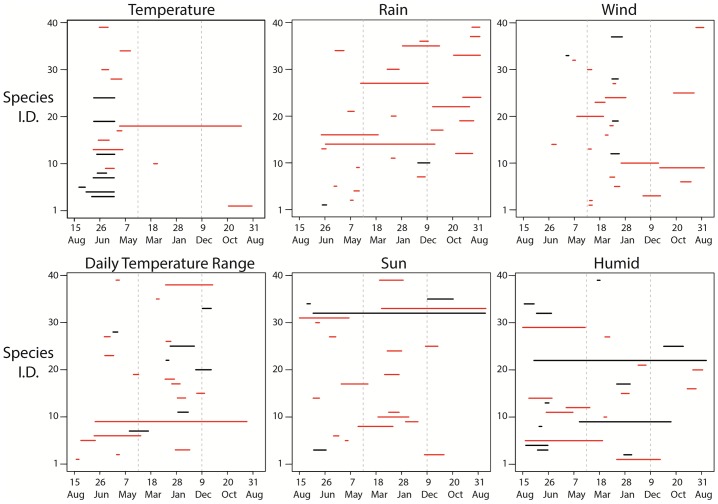
The time periods (or climate windows) over which climatic variables affected body condition. Windows can potentially start from the 15^th^ of August back 365 days before. Red and black lines show whether the relationship is quadratic or linear, respectively. Specific species names can be seen in Table B in S2 Appendix.

In most bird species (39 of the 46), body condition (corrected for size) responded to at least one climate variable, with 34 species affected by multiple climate variables. No particular climate variable was clearly more important overall: out of 46 species, 19 were affected by temperature, 26 by rainfall, 21 by sunshine, 24 by humidity, 25 by wind and 24 by DTR (Fig 3). Temperature was associated with body condition in most species from early May to mid-July (spring-early summer), while wind speed had an effect around early January to mid-April (winter; Fig 3). The time periods for all other climate variables showed much less consistency. The majority of species showed non-linear relationships between body condition and climate: 50% of species showed quadratic responses to humidity, 57% to temperature, 71% to DTR, 80% to wind, 81% to sun and 92% to rain (Fig 3). The average R^2^ value from the final model averaged across all species was 0.19 (1^st^ quartile: 0.14, 3^rd^ quartile: 0.24).

**Fig 4:**
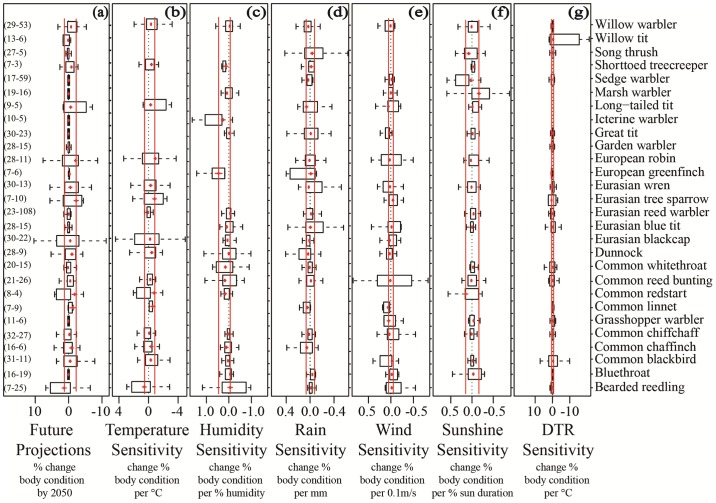
Interspecific and intraspecific variation in future projections (a) and sensitivity (b-g) of 29 bird species at 80 sites. The overall species’ sensitivity (or future projections) are shown by the red points and lines (with standard error bars). The two red vertical lines show the minimum and maximum of the species’ sensitivity estimates (i.e. the range of the red points). For each species, the intraspecific (among-site) variation in climate sensitivity (or future projections) is described by the black boxplots. Sample sizes are shown in brackets along the left side of the figure, with the first number showing how many sites were present followed by the average number of individuals per site per year.

The only climate variables that showed moderately consistent responses in sensitivities across all species were temperature and humidity (Fig 4). Increased temperature was associated with a decrease in body condition for 84% (N = 19) of species (on average -0.4% body condition /°C [95% C.I. = -0.7%, -0.2%]). Increased humidity was associated with increased body condition in 75% (N = 24) of species (on average 0.08% body condition /% humidity [95% C.I. = 0.003%, 0.15%]). Species sensitivities to each climate variable were not strongly correlated, as species that were highly sensitive to one climate variable were not likely to be highly sensitive to any other variables (max.|r| = 0.52; Figure B in S2 Appendix).

Sensitivities also varied substantially within species, with responses at different sites often ranging from positive to negative (Fig 4). Only a few species showed consistent responses among sites for specific climate variables. For instance, almost all sites of the European greenfinch *Carduelis chloris*, icterine warbler *Hippolais icterina* and short-toed treecreeper *Certhia brachydactyla* showed positive responses to humidity.

### Future projections

**Fig 5:**
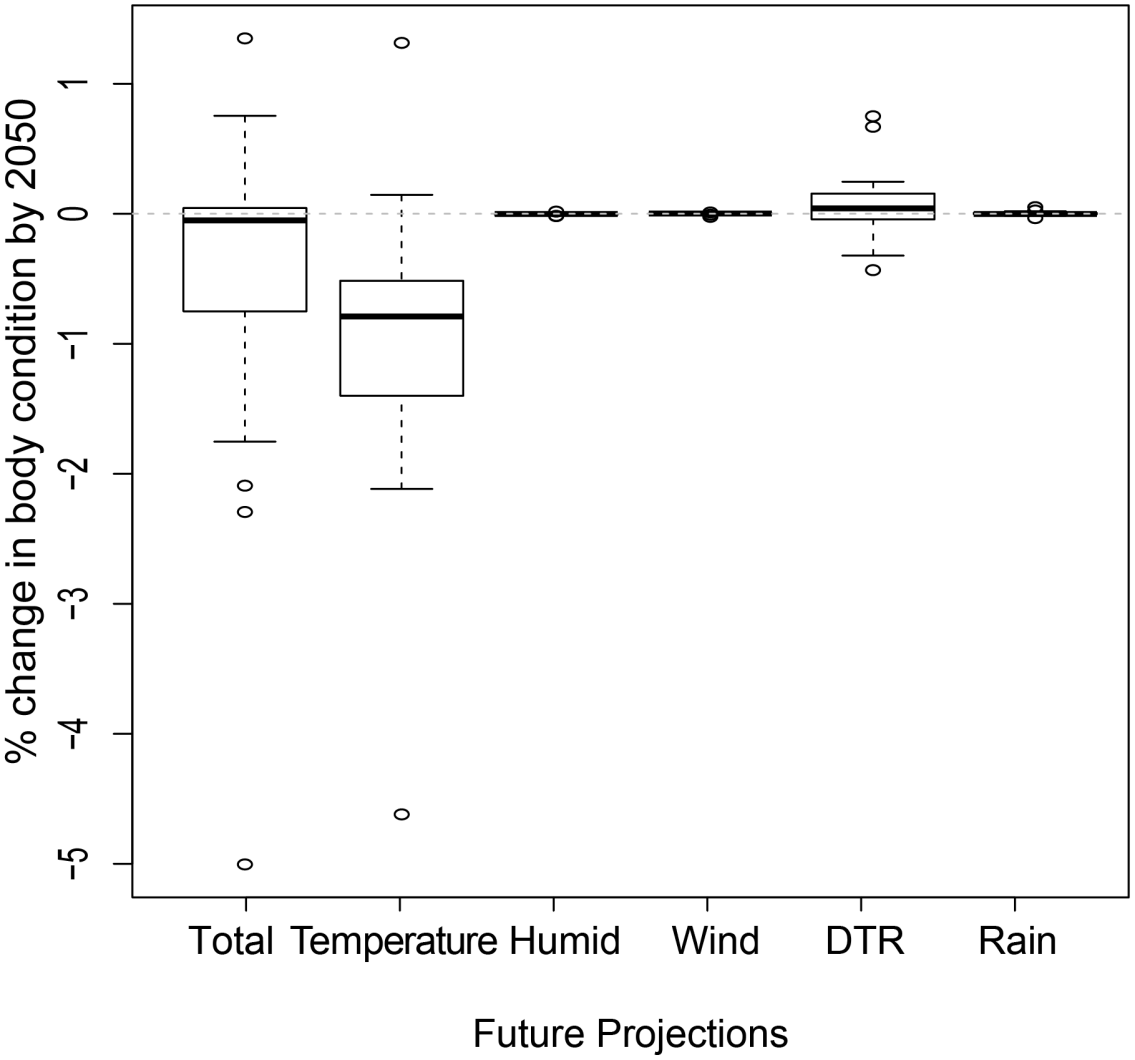
Boxplot of the projected change in percent body condition by 2050 (total future projections) and the contribution of each climate variable for all species for 39 passerine species. Total future projections is the sum of all climate projections.

We found that 62% (N = 39) of species are projected to decrease in body condition by 2050 due to the combined effect of all climate variables. Future projections ranged from 5% decreases to 1.3% increases in body condition by 2050 across all species. However, on average, total body condition is projected to decrease (0.4% ±0.2 SE). Although future projection estimates were made up of sensitivities and exposures to all climate variables, they were mainly driven by temperature (Fig 5), as overall projections were highly correlated to temperature projections (r = 0.98). The projected changes in body condition due to other climate variables were all small, potentially due to smaller changes in these climate variables by 2050 compared to temperature.

### Species signal

Intra-specific variation (for both sensitivity and future projections) was high, such that there was little evidence for any species signals in body condition responses to climate. Visually, population sensitivities within one species varied more than species sensitivities (Fig 4). The variance component models supported this, as the ratio of among-species variation to total variation was low for sensitivity (on average 0.3% across all climate variables, ranging between 0–1.6%) and future projections (0.0%; Table D in S2 Appendix). This suggests that among-population variation was much higher than among-species variation. Such low species variance components mean that the correlation between the climate sensitivity (or future projections) of populations of the same species was no higher than the correlation between populations of different species. Indeed, population sensitivities often differed in their sign, meaning that populations of the same species often showed opposite responses (Fig 4).

### Species and site traits

**Fig 6:**
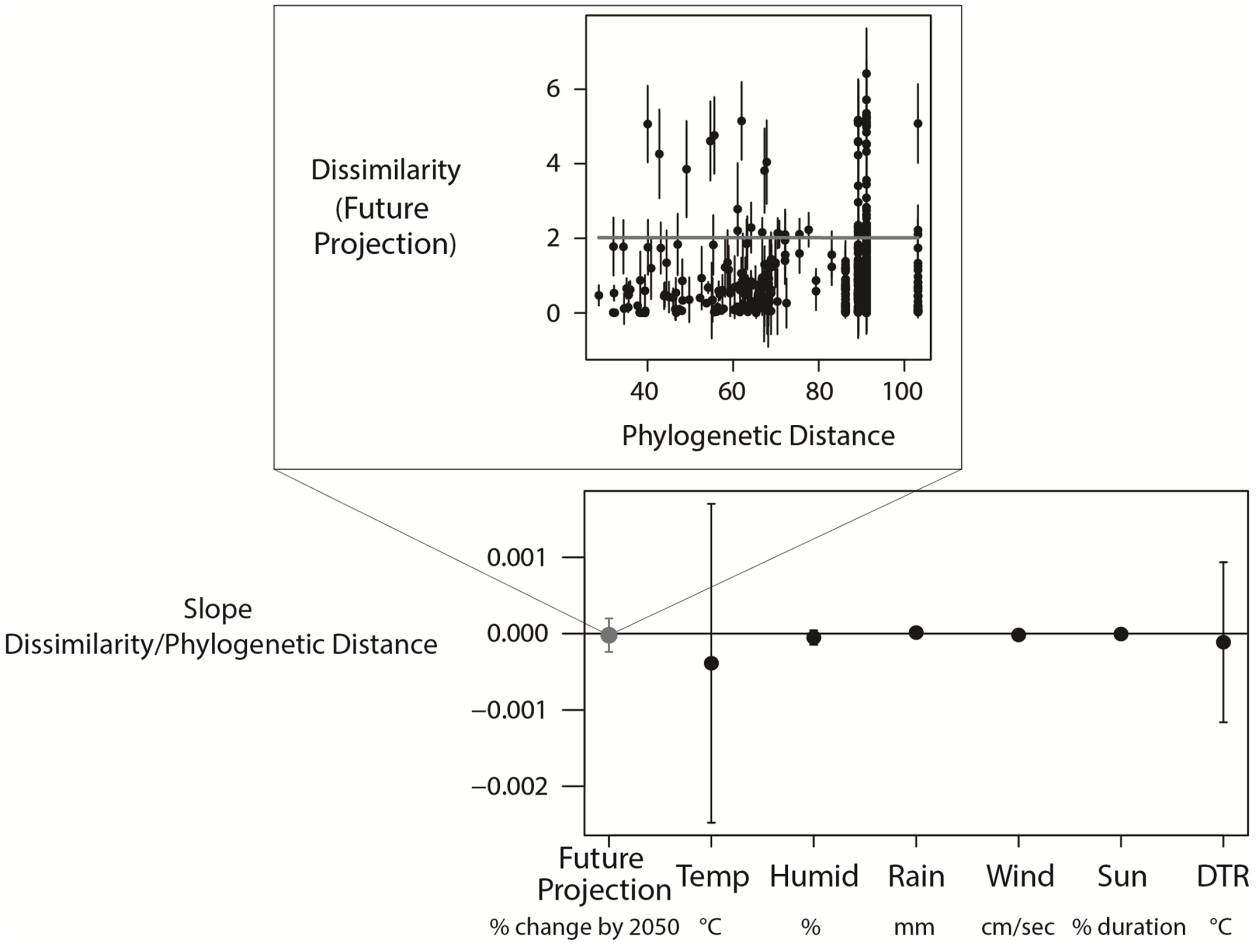
The effect of phylogenetic relatedness on the dissimilarity in future projections and sensitivity of avian body condition. The top figure illustrates what the slopes in the lower figure (the y-axis) represent. In the top figure, we specifically show the relationship between dissimilarity and phylogenetic distance for future projections (grey line), where the slope of 0 reflects that each species had the same projections. A positive slope would indicate that more closely related species have more similar responses of body condition to climate. The bottom figure summarises the slopes obtained from the linear regressions (slope±SE) of phylogenetic relatedness on the dissimilarity in climate sensitivity to each of the six specific climate variables and to future projections (where the grey dot relates to the grey slope in the top figure).

While species traits did not explain variation in species’ sensitivities and future projections, some variation was explained by site-level traits. Variation among species in their climate sensitivity or future projection was not explained by the species traits average body size, migration strategy, preferred habitat type or lifespan (see Tables D & F in S2 Appendix for model selection tables), nor by our proxy for unknown species traits, phylogenetic distance between species (Fig 6). Additionally, species that were more closely related did not tend to be affected by the same climate variables (Figure C in S2 Appendix).

**Fig 7:**
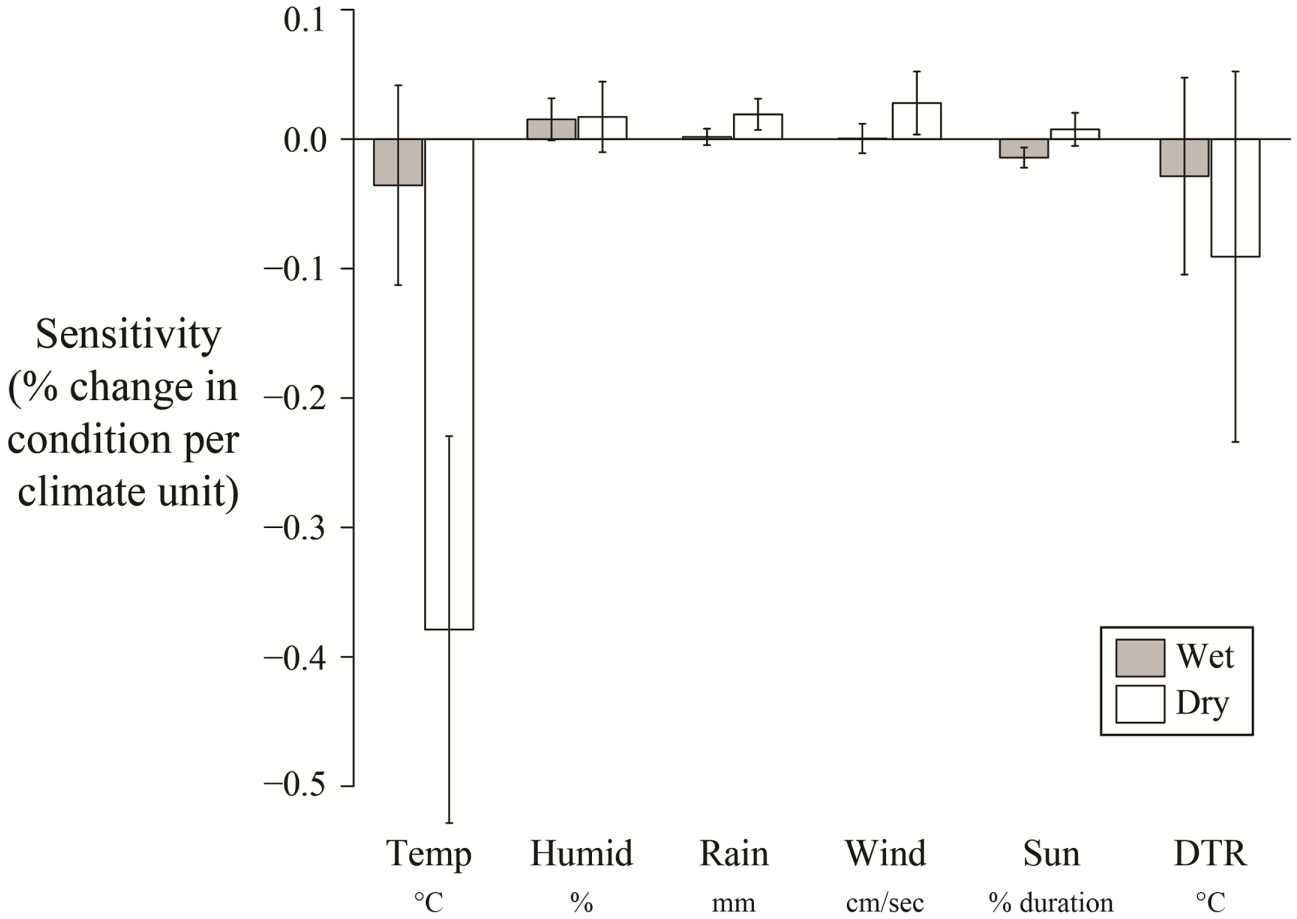
The relationships between site habitat characteristics and sensitivities (±SE) for each climate variable. The shaded bars indicate wet habitats and the clear bars represent dry habitats. Note that the units for each climate variable are not comparable as the units differ.

The site specific trait, habitat type (wet or dry), explained variation in sensitivities to temperature and rainfall among sites (Fig 7). Warmer temperatures in dry habitats resulted in far stronger decreases in body condition compared to populations in wet habitats (which make up 66% of sites; Fig 7). Populations in dry habitats showed comparatively larger increases in body condition per mm of rainfall compared to those in wet habitats (although this model was only slightly better than the null model; Table G in S2 Appendix). Habitat type did not explain variation in future projections, nor did our proxy for habitat quality, population density, explain variation in future projections or sensitivity across sites for any climate variables except humidity.

**Fig 8:**
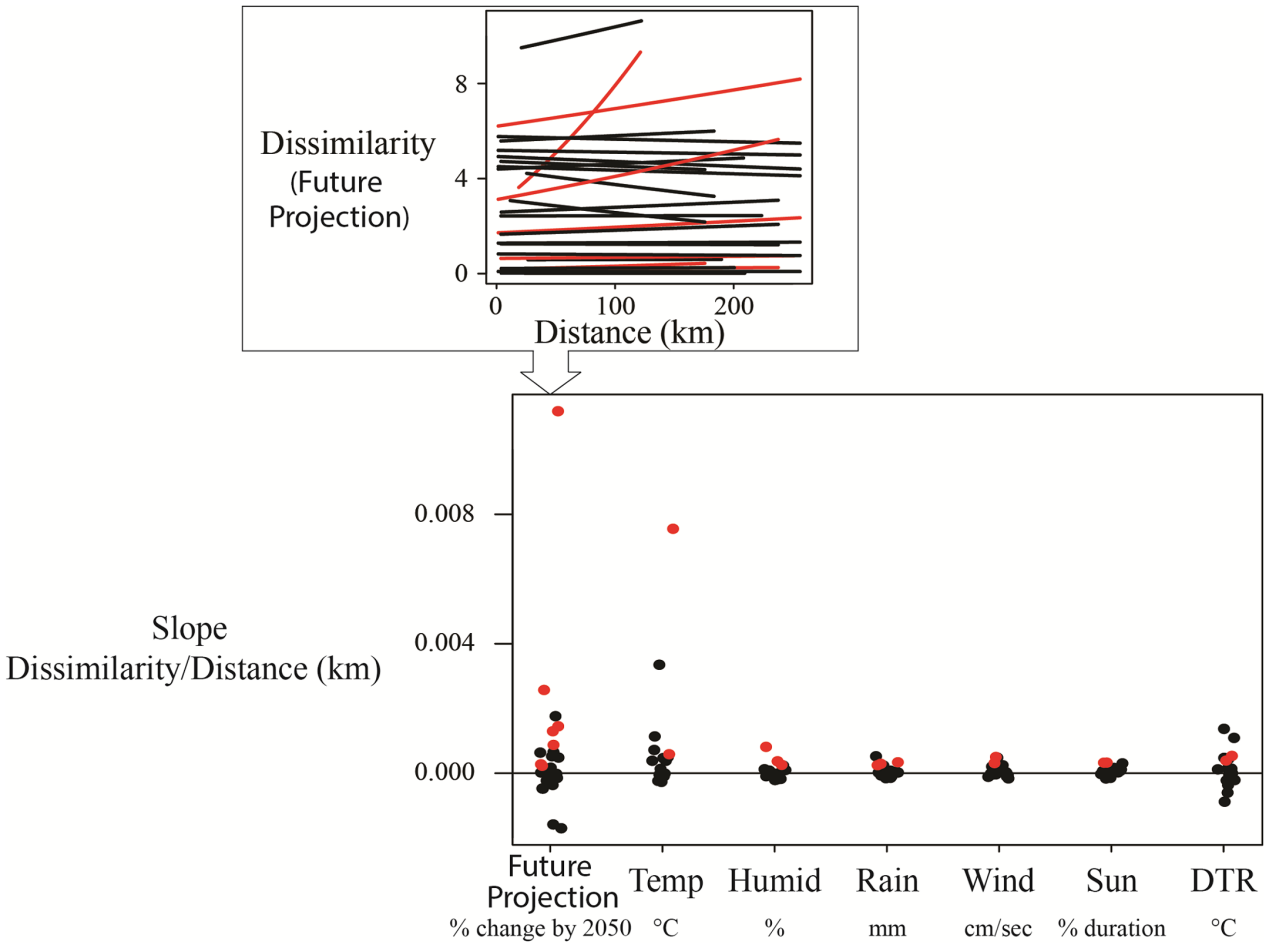
The distribution across species of relationships between distance (km) and dissimilarity in sites sensitivities and future projections. The top figure illustrates how the slopes were estimated in the lower figure (the y-axis). In the top figure, we specifically show the relationship between dissimilarity and distance (km) for future projections, where each line represents a different species. A slope of 0 would mean that projections did not differ with distance, while a positive slope would indicate that closer sites were more similar. The red slopes indicate when a slope was positive and their 95% CI did not cross zero. The bottom figure summarises the slope estimates for each species for future projections and climate sensitivity for each of the six specific climate variables. There were 7 species that showed a significant increase for future projections, 3 species were sensitive to rain, and 2 were sensitive to all other climate variables.

In only a few species, spatial proximity of sites were able to predict dissimilarity in climate sensitivity, with 13% (14 out of 104 species and climate combinations) of species having more similar sensitivities in populations that were closer together (Fig 8; Table H in S2 Appendix). However, future projections were more similar in populations that were closer together for 24% (7 out of 29) of species (Fig 8).

## Discussion

In this study, we aimed to determine how avian body condition responds to changes in climate and how consistent these responses are across, and within, species. The size-adjusted body condition of Dutch birds was sensitive to multiple climate variables, with each of the six climate variables affecting about half of the species, predominantly in a non-linear way. Warmer temperatures were associated with decreased body condition, and higher humidity with increased body condition. However, responses to other climate variables varied widely in direction and size among both species and populations. In the future, body condition was projected to decline in the majority of species, primarily due to temperature effects. We found that sensitivities and future projections among populations of the same species were just as variable as responses across species, suggesting that there was no species signal in climate responses of avian body condition. As a predicted consequence, species traits were unable to explain variation in responses across species, while the local population traits habitat type and geographic distance could explain some of the large amount of among-site variation in climate sensitivity.

### Species signal

By comparing intra-specific variation in body condition sensitivity across 80 populations to inter-specific variation among 39 species, we were able to quantify what ‘a lot’ of intraspecific variation might be. We showed that populations of a given species were almost no more alike than populations of different species in their climate responses, suggesting that species signals in body condition responses likely do not exist here. As such, looking for responses to climate change at a species-specific resolution could be too coarse. Instead, more focus should be on explaining variation in responses within-species. In contrast to our result, Rubolini et al. [5] and Thackeray et al. [7] found that phenological responses over time varied much more across-species than within-species. Yet a study on plant growth response to climate by Malyshev et al. [6], as well as other studies unrelated to climate, show that intraspecific variation can exceed interspecific variation (dispersal ability of butterflies, [8]; range of traits of freshwater fish, [11]). An important question therefore seems to be, why is there a species signal in some response types, such as phenology, but not in others? Only with more research into when such species signals occur for a range of different types of climate responses will we be better able to generate and test potential explanations.

### Species and site traits

Understanding more about how climate change responses vary across populations and species and which species and populations are most at risk is vital. This knowledge will not only help to identify the underlying mechanisms, but is also important for improving the accuracy of our predictions [6], such as those from climate change vulnerability assessments [25,69]. A weak species signal suggests that species-specific traits will be of little use in explaining responses to climate [5]. As such, species traits (including our proxy for unknown traits, phylogenetic distance) did not explain variation in body condition sensitivities to climate or future projections. The current focus in the literature on identifying species life history traits that can explain climatic responses (e.g. [1–3]), is potentially misplaced if the amount of intra-specific variation has not been quantified (as is the situation most of the time) and the average species response is unrepresentative of most individual population responses [5].

The fact that responses varied among sites indicates that local external factors are important (e.g. micro-refugia, habitat quality, resource availability), or that populations themselves may differ in their responses (due to genetic, behavioural or plastic differences). For instance, there was no temperature effect on body condition in wet habitats, suggesting that the overall effect of temperature seems to have been driven by dry habitats. Wet habitats might lessen the direct effects of hot weather through hydration or the opportunity for bathing in available standing water, as populations in dry habitats showed much stronger declines in body condition in hot temperatures than those in wet habitats [70,71]. Alternatively, the effects could work indirectly through food availability. Food abundance fluctuates more strongly in woodland and scrub habitats with warmer temperatures (i.e. dry habitats), as they tend to be less stable than marshes (i.e. wet habitats) [72]. Aubry et al. [28] also found habitat type to be an important predictor of body condition sensitivity to temperature, suggesting that it may more generally have an important role in modulating body condition responses to climate.

Substantial intra-specific variation in responses to environmental change could have important ecological effects [10]. If declining body condition has negative consequences for survival and population growth, for example, then in line with the portfolio effect, variation in body condition responses among populations could counteract the overall effects at higher levels. Indeed, species distribution models show much less severe projections when they take intraspecific variation into account [22]. Somero [73] suggested that populations could be locally adapted, with different genetically determined thermal optima and tolerance limits (see also [74]). If such population variation is heritable this could provide a potential buffer against species extinction [23]. Consequently, the degree of intraspecific consistency has important implications for predicting future projections of species to climate change [5].

### Is temperature the main climatic driver?

In line with previous studies [28–30,34,41], we have shown that warmer temperatures generally resulted in decreased body condition. Temperature was found to be affecting body condition during spring, suggesting that cold winters are not important for body condition during the breeding season for these species living in the temperate climate of the Netherlands. Additionally, we have shown that higher humidity generally resulted in higher body condition. However, as this has not been investigated previously, other studies are needed to establish whether this is a general pattern in birds.

The majority of the literature—on climate change ecology generally, and on body size in particular—only investigates the effects of temperature, yet we found that all six climate variables were important. However, with the exception of temperature and humidity, there were no clear trends across species and the time periods that were associated with the strongest changes in body condition differed substantially (with the exception of temperature and wind speed). Therefore, despite the fact that many species are sensitive to changes in these climate variables, their responses differ substantially. Even populations of the same species differ substantially. It is possible that these associations are not real. However, given how conservative our method to avoid false-positives was and the fact that so many species show associations we do not believe this is likely. Rather, it is possible that only once we have a better understanding of the underlying mechanisms will we be able to tease apart these different responses. In the same way that populations in dry habitats showed different responses to temperature, other unknown local factors could be further influencing these patterns.

Despite all six climate variables being found to be important climatic drivers of body condition, from a future projections perspective to climate change, temperature was found to be the key variable. By combining the sensitivity estimates of species and populations with exposure (i.e. add the change in mass per climate unit with the projected change in climate) we were able to project for the first time how avian body condition might change in the near future. Most species were projected to decrease their body condition by 2050, driven mainly by temperature effects. All other climate variables were less important in determining species’ future projections either because they were less sensitive to these climate variables, or because these variables are projected to change less strongly than temperature. Overall, the current focus in the literature on temperature may actually not be as problematic as first thought, despite the fact that body condition may be sensitive to other climate variables.

### Statistical issues

We performed the first comparative study on body condition responses that investigated multiple climate variables and allowed different time periods to impact different species in linear or non-linear ways. This high level of detail introduces a level of complexity that restricts options for integrative analyses and also makes interpretation more difficult. However, it also includes biological realism that other studies might be missing. For instance, we found that the majority of responses to climate variables were non-linear, and different species were found to be impacted by different combinations of climate variables. The fact that body condition was affected by different climate variables and that different species exhibited different response curvatures meant that the analysis comparing the within- versus among-species variation was not able to be carried out in one single combined analysis based on the individual observations. Instead, we first estimated the climate sensitivity of each population for each species separately and subsequently analysed the climate sensitivity of all populations and species in a subsequent model. Such multi-step procedures (see Fig 1) are not ideal as they introduce issues with propagation of uncertainty (e.g. due to sampling variance among populations), which we tried to alleviate by weighting climate sensitivities by their standard errors and checking for dependencies of their variance on sample size.

Although we took several steps to avoid false positives in our climate window selection, it is possible that some windows could still be identified as the best model by chance. Such false windows could disrupt the detection and explanation of among-species variation. This is inevitable in any study that compares multiple models and species, as all analyses that test a high number of models will face this problem. However, many studies do not even consider the possibility that there is no climate signal, nor do they account for multiple testing of the many potential windows tried [44].

We investigated the local climatic conditions for all migratory and non-migratory species, which could potentially mean that windows were selected during times which migratory species may not be present. In such cases, local conditions could be correlated with their overwintering habitats, for instance if local weather reflects wide-spread conditions due to large-scale oceanic climate indices such as the North Atlantic Oscillation Index [75]. If this is not the case, it is more likely that species sensitivities are occurring via climate effects on habitat quality in the breeding areas. However, the migration strategy of the different species did not explain any differences in sensitivities, suggesting that perhaps the underlying mechanisms acting on body condition do not differ drastically among migratory and non-migratory species.

## Conclusions

The fact that body condition sensitivities to climate varied so substantially among populations of the same species draws attention to the need for researchers to investigate variation within species, and not just to assume that a species-level response will be representative. Given that there are now two studies showing weak species signals in body condition, mass or growth ([6] and this study) and two studies showing strong species signals for phenological changes with climate [5,7], other comparative analyses are needed to better understand how frequently species signals are occurring and how this may vary among traits. This is particularly needed for other key traits such as physiology or offspring sex ratio, or vital rates such as survival and reproduction. The absence of a species signal in the climate sensitivity of vital rates determining population growth, for instance, would be problematic for conservation prioritisation. Climate change vulnerability assessments based solely on species traits would be inadequate, while intraspecific traits such as habitat type might be more useful. Accurate predictor variables need to be identified if we are to improve conservation management planning, especially given that almost all species were projected to decrease in body condition by 2050.

## Supporting information

S1 Appendix Supporting information for methods and materials. (DOC)

S2 Appendix Supporting information for results. (DOCX)
