# Supplementary material for: High intra-specific variation in avian body condition responses to climate limits generalisation across species
Source: PLoS One. 2018 Feb 21;13(2):e0192401. doi: 10.1371/journal.pone.0192401 (PMC5821336; doi:10.1371/journal.pone.0192401)
Supplement: S1 Appendix — (DOC) [file pone.0192401.s001.doc]

**Appendix S1: Methods**

**1. Data**

1.1 Body condition data for common Dutch bird species 1

1.2 Climatic data 2

1.3 Species and site trait data 4

**2. Statistical analyses**

2.1 Climate windows and signals 5

2.2 Reducing False Positives 6

2.3 Model Selection and Averaging 9

2.4 Age and Sex Differences 11

2.5 Climate Sensitivities 12

2.6 Climate Vulnerabilities 13

2.7 Species and site traits 13

# 1. Data

1.1 Body condition data for common Dutch bird species


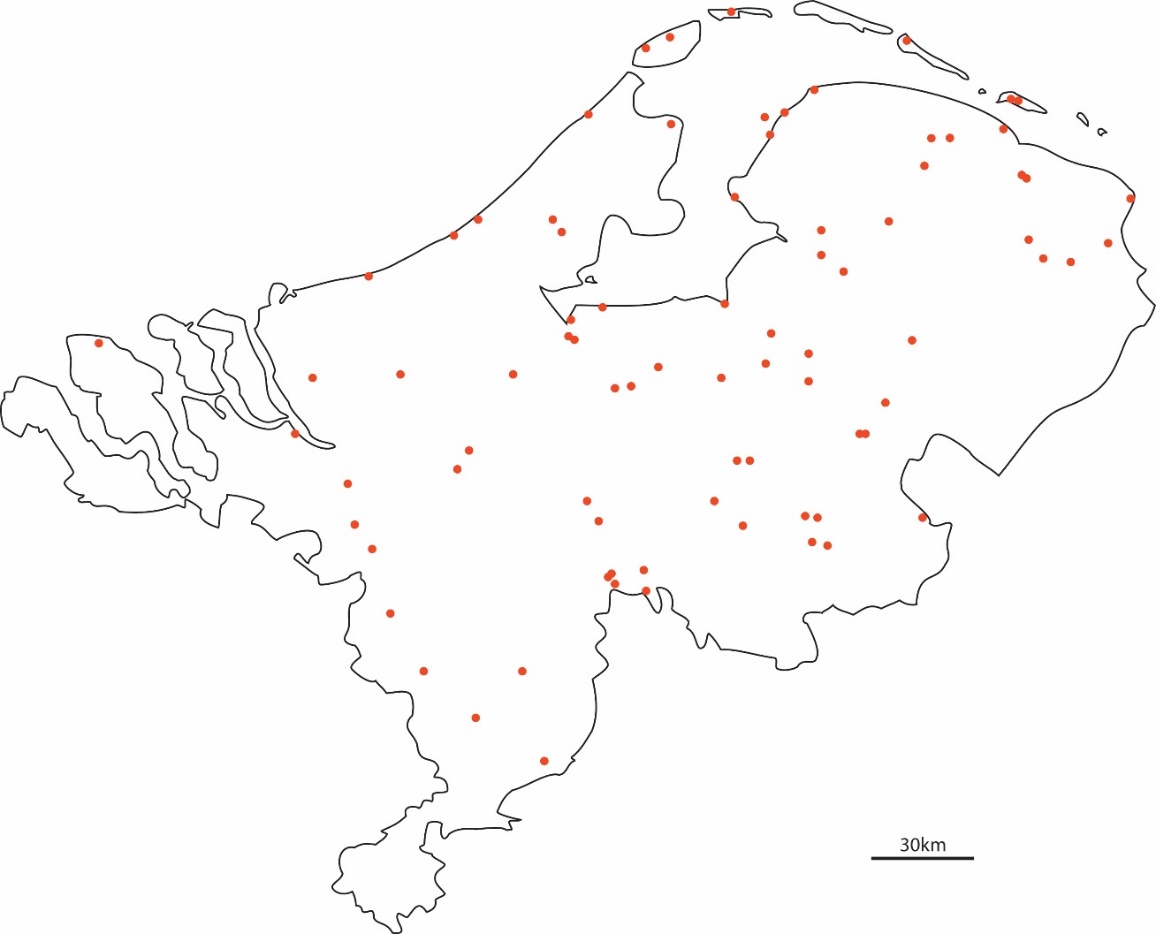


Fig A. Map of the Netherlands with all study sites (indicated by the red dots).

**Data selection.** For data selection we removed observations in which (i) measurements seemed highly likely to be mistakes, (ii) the bird had died, and (iii) crucial parameters were not recorded (i.e. missing body mass, wing length, date of capture, or age-class). Before conducting any of our analyses, we first removed any extreme values that we deemed as highly likely to be mistakes in the data (e.g. typos; it was not uncommon to find values orders of magnitude out from the main distribution). We used a two-step process to remove any suspicious data points in a standardised way. Firstly, for each species, we calculated where the inner 68.27% of the data lay from the median (approximately 1 standard deviation; i.e. 34.13% of the data on either side of the median) and then extrapolated in both directions to 3 standard deviations (99.7%). Any data points that were outside of this 99.7% region were considered extreme values and were removed. This method of identifying extreme values is preferable to using the mean and standard deviation, since it is unaffected by the extreme values or skews in the distribution. Secondly, we conducted a regression with body mass as the response variable and wing length as the explanatory variable. Any residuals that were outside of 3 standard deviations (calculated in the same way as above) were removed. This step identified any potential mistakes that would not have been recognised independently, for instance, a bird with small wing length that is very heavy or large wing length that is extremely light. Although it is possible that we could have potentially removed real values, we decided that it was more important to be certain that those values that we do use in our analysis were real. Several species did not have any data collected in the year 2002.

Table A. Species removed due to low sample sizes. These species’ sample sizes were too small for the model to converge in the R package *climwin.*

| **Common Name** | **# Individuals** | **Years** |
| --- | --- | --- |
| Barn swallow | 638 | 20 |
| Coal tit | 120 | 14 |
| Common redpoll | 53 | 7 |
| Eurasian penduline tit | 105 | 15 |
| Great reed warbler | 65 | 19 |
| Meadow pipit | 151 | 17 |
| Sand martin | 16 | 8 |
| Western yellow wagtail | 35 | 9 |

1.2 Climatic data

Table B. CES site and weather station coordinates and the distances between the two.

| **CES Site** | **Lat (CES)** | **Long (CES)** | **Weather Station** | **Lat (Stn)** | **Long (Stn)** | **Distance (km)** |
| --- | --- | --- | --- | --- | --- | --- |
| C01 | 52.61 | 5.90 | 273 | 52.70 | 5.89 | 10.1 |
| C02 | 52.45 | 5.82 | 269 | 52.46 | 5.53 | 20.3 |
| C03 | 52.34 | 4.52 | 240 | 52.30 | 4.77 | 17.7 |
| C04 | 52.54 | 6.47 | 278 | 52.44 | 6.26 | 18 |
| C05 | 53.21 | 5.44 | 270 | 53.23 | 5.76 | 21.2 |
| C06 | 53.11 | 4.79 | 235 | 52.92 | 4.79 | 20.3 |
| C07 | 53.20 | 6.80 | 280 | 53.13 | 6.59 | 16.8 |
| C08 | 52.33 | 5.16 | 265 | 52.13 | 5.27 | 23.7 |
| C09 | 52.91 | 5.83 | 273 | 52.70 | 5.89 | 23.9 |
| C10 | 51.85 | 5.12 | 356 | 51.86 | 5.15 | 1.9 |
| C11 | 52.61 | 5.64 | 269 | 52.46 | 5.53 | 18.2 |
| C12 | 51.57 | 4.90 | 350 | 51.57 | 4.93 | 2.1 |
| C13 | 53.04 | 5.40 | 267 | 52.90 | 5.38 | 15.8 |
| C14 | 52.54 | 4.83 | 249 | 52.64 | 4.98 | 15.6 |
| C15 | 53.26 | 4.95 | 251 | 53.39 | 5.35 | 30.9 |
| C16 | 51.69 | 4.47 | 344 | 51.96 | 4.44 | 29.8 |
| C17 | 52.23 | 6.61 | 283 | 52.07 | 6.65 | 17.3 |
| C18 | 52.28 | 6.52 | 283 | 52.07 | 6.65 | 24.8 |
| C19 | 52.29 | 6.09 | 278 | 52.44 | 6.26 | 20.5 |
| C20 | 52.63 | 6.08 | 273 | 52.70 | 5.89 | 15.5 |
| C21 | 52.42 | 5.23 | 269 | 52.46 | 5.53 | 20.4 |
| C22 | 52.53 | 6.46 | 278 | 52.44 | 6.26 | 16.6 |
| C23 | 51.83 | 5.93 | 375 | 51.66 | 5.71 | 24.5 |
| C24 | 52.85 | 5.44 | 267 | 52.90 | 5.38 | 6.2 |
| C25 | 51.84 | 5.96 | 275 | 52.06 | 5.89 | 25.1 |
| C26 | 52.44 | 6.88 | 290 | 52.27 | 6.90 | 18.5 |
| C27 | 51.34 | 5.79 | 377 | 51.20 | 5.76 | 16.1 |
| C28 | 52.42 | 4.56 | 240 | 52.30 | 4.77 | 19.7 |
| C29 | 51.44 | 5.24 | 370 | 51.45 | 5.41 | 12.4 |
| C30 | 51.84 | 4.39 | 344 | 51.96 | 4.44 | 13.4 |
| C31 | 52.14 | 4.33 | 210 | 52.17 | 4.42 | 6.8 |
| C32 | 52.31 | 5.21 | 265 | 52.13 | 5.27 | 20.5 |
| C33 | 53.33 | 6.42 | 277 | 53.41 | 6.20 | 16.9 |
| C34 | 53.07 | 5.33 | 267 | 52.90 | 5.38 | 19.4 |
| C35 | 52.86 | 6.00 | 273 | 52.70 | 5.89 | 19.1 |
| C36 | 52.31 | 6.13 | 278 | 52.44 | 6.26 | 16.9 |
| C37 | 51.57 | 3.57 | 310 | 51.44 | 3.60 | 14.2 |
| C38 | 52.27 | 6.47 | 278 | 52.44 | 6.26 | 23.7 |
| C39 | 53.04 | 4.74 | 235 | 52.92 | 4.79 | 13.2 |
| C40 | 51.65 | 4.77 | 350 | 51.57 | 4.93 | 14.7 |
| C41 | 53.33 | 6.43 | 277 | 53.41 | 6.20 | 17.8 |
| C42 | 52.81 | 6.42 | 279 | 52.75 | 6.58 | 12.6 |
| C43 | 52.91 | 5.03 | 235 | 52.92 | 4.79 | 16.3 |
| C44 | 52.86 | 5.88 | 273 | 52.70 | 5.89 | 17.5 |
| C45 | 53.31 | 6.05 | 277 | 53.41 | 6.20 | 14.7 |
| C46 | 53.46 | 5.66 | 251 | 53.39 | 5.35 | 21.8 |
| C47 | 53.21 | 6.03 | 270 | 53.23 | 5.76 | 18.8 |
| C48 | 51.90 | 5.12 | 356 | 51.86 | 5.15 | 4.9 |
| C49 | 52.54 | 5.95 | 273 | 52.70 | 5.89 | 18.7 |
| C50 | 52.57 | 6.15 | 278 | 52.44 | 6.26 | 16.9 |
| C51 | 51.35 | 6.13 | 391 | 51.50 | 6.20 | 17.4 |
| C52 | 51.36 | 5.49 | 370 | 51.45 | 5.41 | 11.2 |
| C53 | 52.14 | 5.09 | 260 | 52.10 | 5.18 | 7.6 |
| C54 | 53.20 | 6.59 | 280 | 53.13 | 6.59 | 8 |
| C55 | 52.27 | 5.48 | 265 | 52.13 | 5.27 | 20.9 |
| C56 | 51.97 | 4.69 | 348 | 51.97 | 4.93 | 16.4 |
| C57 | 53.44 | 6.87 | 286 | 53.20 | 7.15 | 32.6 |
| C58 | 53.41 | 6.22 | 277 | 53.41 | 6.20 | 1.9 |
| C59 | 52.22 | 6.55 | 283 | 52.07 | 6.65 | 17.5 |
| C60 | 51.51 | 5.84 | 375 | 51.66 | 5.71 | 18.8 |
| C61 | 52.33 | 5.16 | 265 | 52.13 | 5.27 | 23.7 |
| C62 | 53.48 | 6.16 | 277 | 53.41 | 6.20 | 8.3 |
| C63 | 51.91 | 6.03 | 275 | 52.06 | 5.89 | 20 |
| C64 | 51.87 | 6.08 | 275 | 52.06 | 5.89 | 25 |
| C65 | 51.96 | 5.74 | 275 | 52.06 | 5.89 | 15.4 |
| C66 | 52.52 | 4.91 | 249 | 52.64 | 4.98 | 14.8 |
| C67 | 52.53 | 6.15 | 278 | 52.44 | 6.26 | 12.7 |
| C69 | 52.42 | 5.23 | 269 | 52.46 | 5.53 | 20.3 |
| C70 | 52.29 | 5.53 | 269 | 52.46 | 5.53 | 18.3 |
| C71 | 53.28 | 6.00 | 270 | 53.23 | 5.76 | 17.4 |
| C72 | 51.82 | 5.94 | 375 | 51.66 | 5.71 | 24.2 |
| C73 | 53.17 | 6.70 | 280 | 53.13 | 6.59 | 9 |
| C74 | 52.37 | 5.58 | 269 | 52.46 | 5.53 | 10.4 |
| C75 | 52.81 | 4.69 | 235 | 52.92 | 4.79 | 14.3 |
| C76 | 53.04 | 6.04 | 270 | 53.23 | 5.76 | 28.6 |
| C77 | 53.31 | 6.89 | 286 | 53.20 | 7.15 | 21.5 |
| C78 | 52.57 | 6.14 | 278 | 52.44 | 6.26 | 16.8 |
| C79 | 52.16 | 6.26 | 275 | 52.06 | 5.89 | 27.6 |
| C81 | 51.55 | 5.03 | 350 | 51.57 | 4.93 | 7 |
| C82 | 53.48 | 6.18 | 277 | 53.41 | 6.20 | 8.4 |
| C83 | 52.63 | 6.47 | 279 | 52.75 | 6.58 | 14.7 |
| C84 | 53.44 | 6.87 | 286 | 53.20 | 7.15 | 32.6 |
| C85 | 52.31 | 5.20 | 265 | 52.13 | 5.27 | 20.6 |
| C86 | 52.17 | 6.10 | 275 | 52.06 | 5.89 | 18.9 |
| C87 | 51.98 | 5.66 | 275 | 52.06 | 5.89 | 18.2 |
| C88 | 53.10 | 5.39 | 267 | 52.90 | 5.38 | 22.7 |

A small number of the daily measurements at some weather stations were missing; such potential windows were excluded in the climate window analysis.


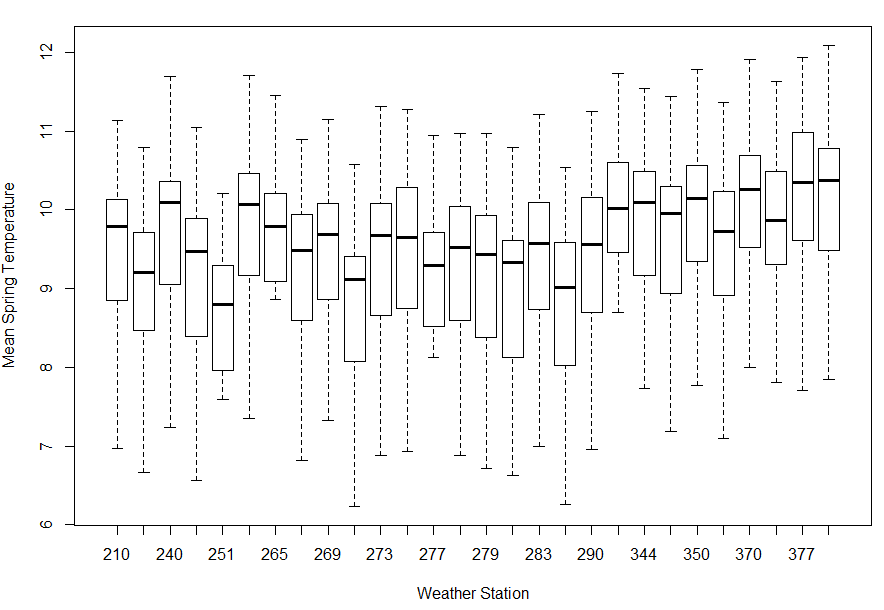


Fig B. Variation in mean spring temperature among the different weather stations. Here, we show annual mean spring temperatures for each weather station over the course of the study period. This shows that temporal variation within sites is larger than the spatial variation among sites.

1.3 Species and site trait data

Adult life-expectancy was calculated as
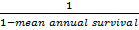
 for the span of the study. Each habitat category (Urban, Woodland, Wet, Open) was coded as a separate dummy variable (0 or 1) because each species could potentially be found in multiple habitat types. Habitats were assigned to each species based on Mullarney et al. (2000), however we condensed the number of potential habitat types in the following way:

- Urban: parks, gardens, farmland, human habitation and orchards
- Woodland: woods, scrubby areas, open woodland, mixed woods, coniferous forest, bushy areas, open forest, deciduous woods and upland birch forest
- Wet: reedbeds, lowland lakes, swampland, rivers and lakes, coastal heaths and wet ground
- Open: heaths and commons

**2. Statistical analyses**

2.1 Climate windows and signals

The R package *climwin* (Bailey & van de Pol 2015) was used to find the time period during which the mean of each of the six climatic variables explained the most variation in body condition for each species. Because we were most interested in identifying the periods over which these climatic variables affected body condition, we were looking for fixed windows (i.e. all individuals were assumed to be affected by climate over the same time period) rather than variable windows (time windows that are relative to the timing of expression or measurement of a trait for each individual) (van de Pol et al., 2016). The *climwin* analysis calculates the mean of the daily climate estimates for each site per year over the specific period being tested (e.g. mean of all days in May) and conducts a linear regression with this mean climate as the predictor variable and body condition as the response variable. The AIC model fit value is used to compare each regression model to a null model without climate (thereby providing an indication of how much better the model with those mean climate estimates is to one without climate at all). This is repeated until all possible combinations of consecutive days have been tested.

We first determined a baseline model structure without climate effects. The baseline model takes into account the age of individuals (juvenile or adult; where juveniles are first years and adults are older than 1 year), a quadratic effect of weighing time (minutes since midnight) and a quadratic effect of the time in the season (number of days since 1^st^ April). Individual ID was included as a random effect to account for non-independence of those birds that were captured multiple times. Unfortunately, we could not include both Site ID and Individual ID as a random effect because most species did not have large enough sample sizes to deal with such a complex model. Individual ID was chosen to remain in the model (rather than Site ID) because it explained more variation. The baseline model was the same for all species with the exception that sex (male, female or unknown) was only included for those species that could be accurately sexed in the field.

Table C. The 12 models analysed for each species using the *climwin* function.

|  | **Model** |
| --- | --- |
| 1 | Body condition ~ mean temperature + baseline |
| 2 | Body condition ~ mean rainfall + baseline |
| 3 | Body condition ~ mean wind speed + baseline |
| 4 | Body condition ~ mean humidity + baseline |
| 5 | Body condition ~ mean % sunshine + baseline |
| 6 | Body condition ~ mean DTR + baseline |
| 7 | Body condition ~ mean temperature^2 + baseline |
| 8 | Body condition ~ mean rainfall^2 + baseline |
| 9 | Body condition ~ mean wind speed^2 + baseline |
| 10 | Body condition ~ mean humidity^2 + baseline |
| 11 | Body condition ~ mean % sunshine^2 + baseline |
| 12 | Body condition ~ mean DTR^2 + baseline |

2.2 Reducing False Positives

When testing so many models, the chances of spurious results are high (van de Pol et al., 2016). We took two steps to reduce the chances of getting false positives (type I errors). Firstly, we excluded short windows that we deemed unlikely to be biologically plausible. Windows that were shorter than 5 days were excluded, and windows less than 15 days for if they were further than 60 days before the sampling season starts (on the 12^th^ April; i.e. long-term windows). Such short windows are biologically less plausible and are often incorrectly selected as best windows by chance (van de Pol et al., 2016).

Secondly, we performed a formal randomisation analysis to determine if climate windows were likely to be false positives. If the randomisation analysis suggested that the best fitted model was likely a true positive, this window was added into the baseline model and the *climwin* analysis was rerun to check whether any second climate signal window was present after accounting for the best window. If it suggested that the best model was likely only due to chance, that species was not considered to show a climate signal, meaning that body condition was not considered to be affected by that climate variable. The randomisation analysis determined a highly conservative cut-off AICc value (see below), above which time windows were considered to be false positives. To quantify the likelihood of obtaining strong model support by chance we randomised the data and analysed the distribution of ‘best’ AICc values. Rather than carrying out multiple randomisations for each species, due to constraints with computational time, we carried out three randomisations on a subset of 15 species (Table D).

Table D. Subset of species selected and their sample sizes (total number of individuals and total number of years captured) used in the randomisation analysis.

|  | **Common Name** | **# Individuals** | **Years** |
| --- | --- | --- | --- |
| 1 | Common blackbird | 5817 | 20 |
| 2 | Common chaffinch | 1511 | 19 |
| 3 | Common chiffchaff | 15089 | 20 |
| 4 | Common grasshopper warbler | 1182 | 20 |
| 5 | Common Kingfisher | 177 | 13 |
| 6 | Common linnet | 969 | 19 |
| 7 | Eurasian jay | 232 | 18 |
| 8 | Eurasian nuthatch | 127 | 17 |
| 9 | European crested tit | 380 | 19 |
| 10 | European goldfinch | 431 | 16 |
| 11 | European pied flycatcher | 358 | 19 |
| 12 | Great tit | 10720 | 20 |
| 13 | Lesser whitethroat | 1594 | 20 |
| 14 | Marsh tit | 299 | 18 |
| 15 | Marsh warbler | 5197 | 21 |

The same *climwin* analyses were carried out three times on the randomised data for each climate variable and for linear and quadratic responses (i.e. 6 climate variables x 2 responses types x 3 replications = 36 randomised analyses per species). The AICc values from the best model were recorded from each randomisation. These values were then used to determine what the AICc cut-off would be, whether this cut-off value should be different for each climate variable, for linear or quadratic responses and for different species (with different sample sizes).

1. Should the AICc cut-off value differ between climate variables?

Using the best model AICc values from the randomised analyses, we found a small difference between the best model AICc values among climate variables (Fig C a). A linear model with climate variables as the explanatory variable and ‘best model AICc’ as the predictor variable was 6.3 AICc units better than the null model (intercept only model). However, the mean best-model AICc only ranged from -7.5 to -5.3 AICc units across the different climate variables, suggesting that although the randomised AICc values did differ among climate variables, this difference was small.


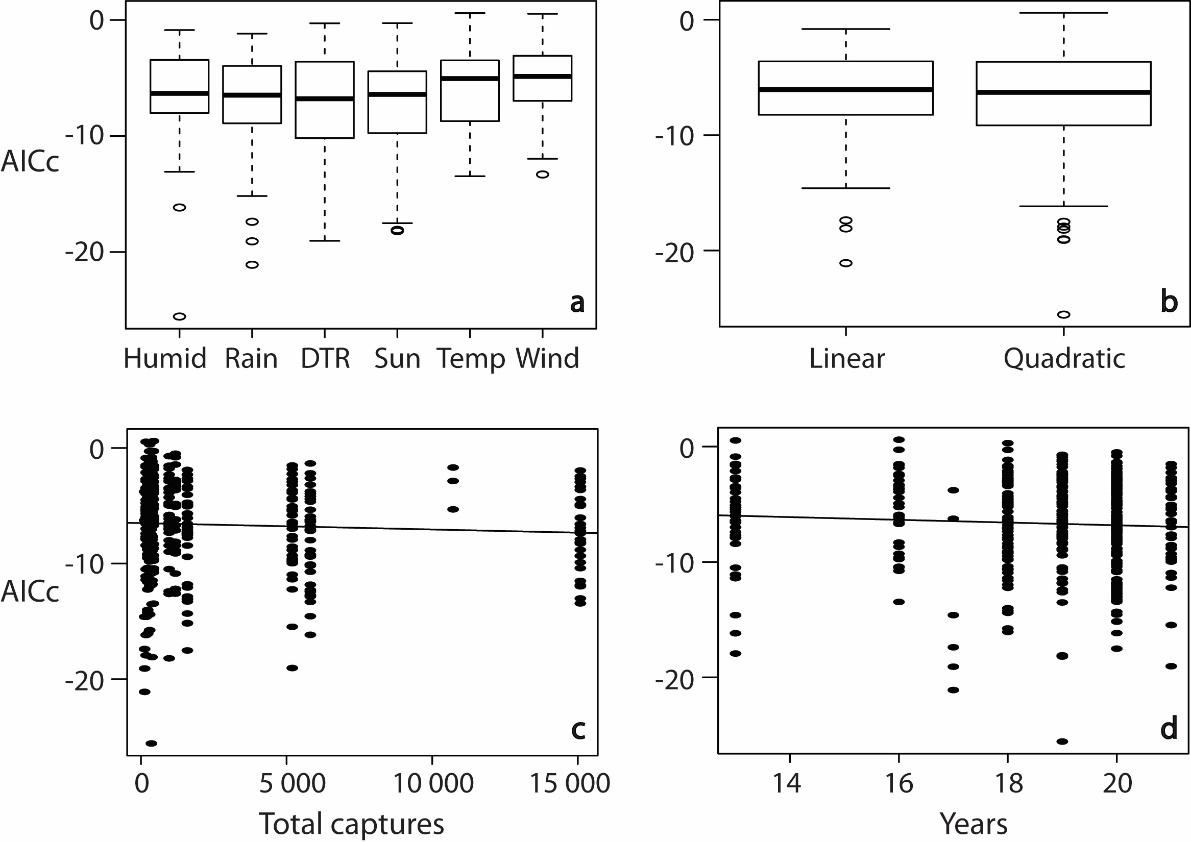


Fig C. Best model delta AICc values from the randomisation analysis. Here, we show an overview of how the best-model AICc values from randomised climwin analyses vary with (a) climatic variables, (b) linear or quadratic responses to climate variables, (c) differing numbers of total captures of individuals, and (d) differing numbers of years with observations.

1. Should the AICc cut-off value differ between linear and quadratic responses in body condition?

We found that there was no difference between the randomised best model AICc values among linear or quadratic response types (Fig C b). A linear model with curvature (linear or quadratic as categorical variables) as the explanatory variable and ‘best model AICc’ as the predictor variable was within 2 AICc units of the null model (intercept only model).

1. Should the AICc cut-off value differ among species with different sample sizes?

Overall sample size (total number of individuals caught; Fig C c) and the number of years (Fig C d) had no effect on the randomised best-model AICc values. Both models were within 2 AICc units of the null model.

As we found little evidence suggesting that the best-model AICc values from the random data differed, we decided to use a single cut-off value for all climate variables, linear and quadratic responses, and species (even over a range of years and sample sizes). The majority of the randomised best model AICc values fell between 0 and -10, with a mean AICc value of -6.6 (Fig D). In order to keep our cut-off value conservative, we chose to assign a value of -14.5. This value was 2 standard deviations lower than the mean (standard deviation = 3.9). This conservative cut-off value also reduces any potential for differences in false positive rates among climate variables. If AICc values from the real analyses were higher than the cut-off value they were not considered a real window. This decision will mean that we will be rejecting some ‘real’ climate windows, but we felt that it was more important to be certain that those windows that we do accept as being correct are as likely as possible to be real (true positives).


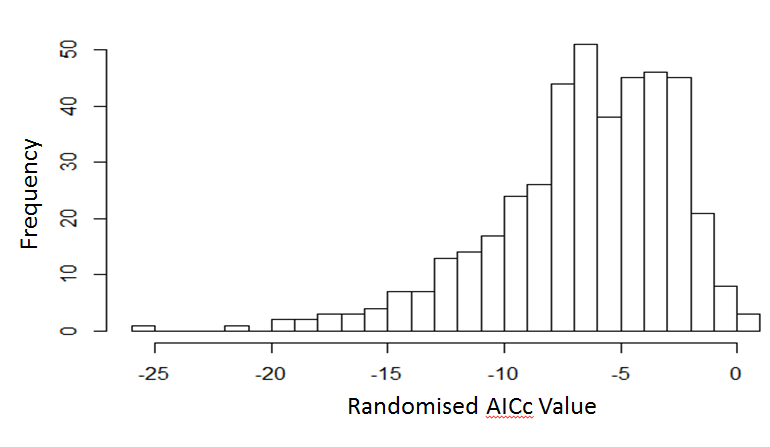


Fig D. Frequency of the best model AICc values from the randomised data across all species and climate variables.

When the best window’s AICc value was below the cut-off value, then the best model was added into the baseline model and the *climwin* analysis was run a second time to check whether any additional windows were present after accounting for the best window. If a second window was found, but the critical time period overlapped with the first window, then we discarded the second window and focused on the first window only. To determine whether the body condition response was linear or non-linear, the model with the best AICc value was selected. However, if the best AICc value was within 2 AICc units of one another then we selected the linear response.

2.3 Model Selection and Averaging

Model selection and averaging was used to determine which climate variables were important and to calculate parameter estimates after accounting for the other climate variables. We adopted an Information-Theoretic approach to quantify and rank the models based on the different climate variables (Burnham and Anderson, 2002). Climate variables were considered hitchhikers and were subsequently removed from the model set if they met two requirements, (1) the addition of the climatic variable did not improve the model by > 2 AICc units (i.e. it does not occur in the best model but does occur in a model that is within 2 AICc units of the very best model) (Arnold, 2010), and (2) if their relative importance was less than 0.5 (w+) (K. Burnham and Anderson, 2002). We used the function ‘importance’ from the package AICcmodavg.

Climate variables were rarely correlated, but when this did occur, collinear climate variables were always kept in the same models when testing the different combinations of variables (i.e. both always included or excluded from the same models)(Freckleton, 2010). This allows the effects of the two climate variables, which are essentially indistinguishable, to be measured together and contrasted with the other models. If one variable was to be removed from the model, we would run the risk of systematically over or under-estimating the remaining variable depending on the sign of the correlation between the predictors (Freckleton, 2010). For example if rainfall and temperature were negatively correlated (hot conditions associated with low rainfall) and low rainfall increased body condition while high temperatures decreased body condition, then removing, say rainfall, would result in an under-estimated slope value of the effect of temperature on body condition (Freckleton, 2010).

Once the final climatic variables had been identified we calculated model-averaged slope estimates for each of the sites (or populations). By combining all of the important climate variables into the final model, we can account for their effects when calculating body condition responses. Model averaging uses the average of the parameter estimates from each candidate model, weighted by its Akaike weight (Freckleton, 2010). The reference categories used for the categorical variables were adult (as opposed to juvenile) and (if the species could be sexed) female (as opposed to male) (see Age and Sex Difference section below). Climate variables were all mean centred. Standardised model-averaged slope estimates were also calculated by dividing the mean centred climate variables by their standard deviation. To calculate the model-averaged slope estimates for each site, an interaction term between the site and the climate variable was included. This analysis was run for every site and climate variable separately to make sure that the number of parameters in the model remained low enough that the model could converge. In this way, sensitivity estimates along with their standard errors for all relevant climate variables were calculated for every site.

2.4 Age and Sex Differences

We investigated whether responses to each of the climate variables differed between adults and juveniles, and males and females. Only 40 of the 181 species by climate variable combinations were found to have an interaction term between age and climate that improved the original model (when looking at the model-averaged slope estimates, not sensitivity values), and 13 of the 83 species by climate variable combinations were found to have an interaction term between sex and climate. This suggests that the majority of responses to climate are similar among sex and age groups. We also calculated the sensitivity estimates (rather than the slopes) of the adults and juveniles, and males and females (by including the interaction term between age or sex and climate). We compared these sensitivity estimates to the original sensitivity estimates (without the interaction) to see how well correlated they were. Adult sensitivities (from the interaction) were extremely strongly correlated to the original sensitivity values, suggesting that there was no overall difference between those values estimated with an interaction term and those without (Fig E a). We also found moderate correlation between the adult and juvenile sensitivities (Fig E b). This suggested that adults and juveniles have very similar responses (i.e. interactions were sometimes significant, but their effect size was weak). The sensitivity estimates of females also closely correlated to the original sensitivities (Fig E c). The correlation between male and female sensitivities was very strong (Fig E d), suggesting that males and females have very similar sensitivities to climate.


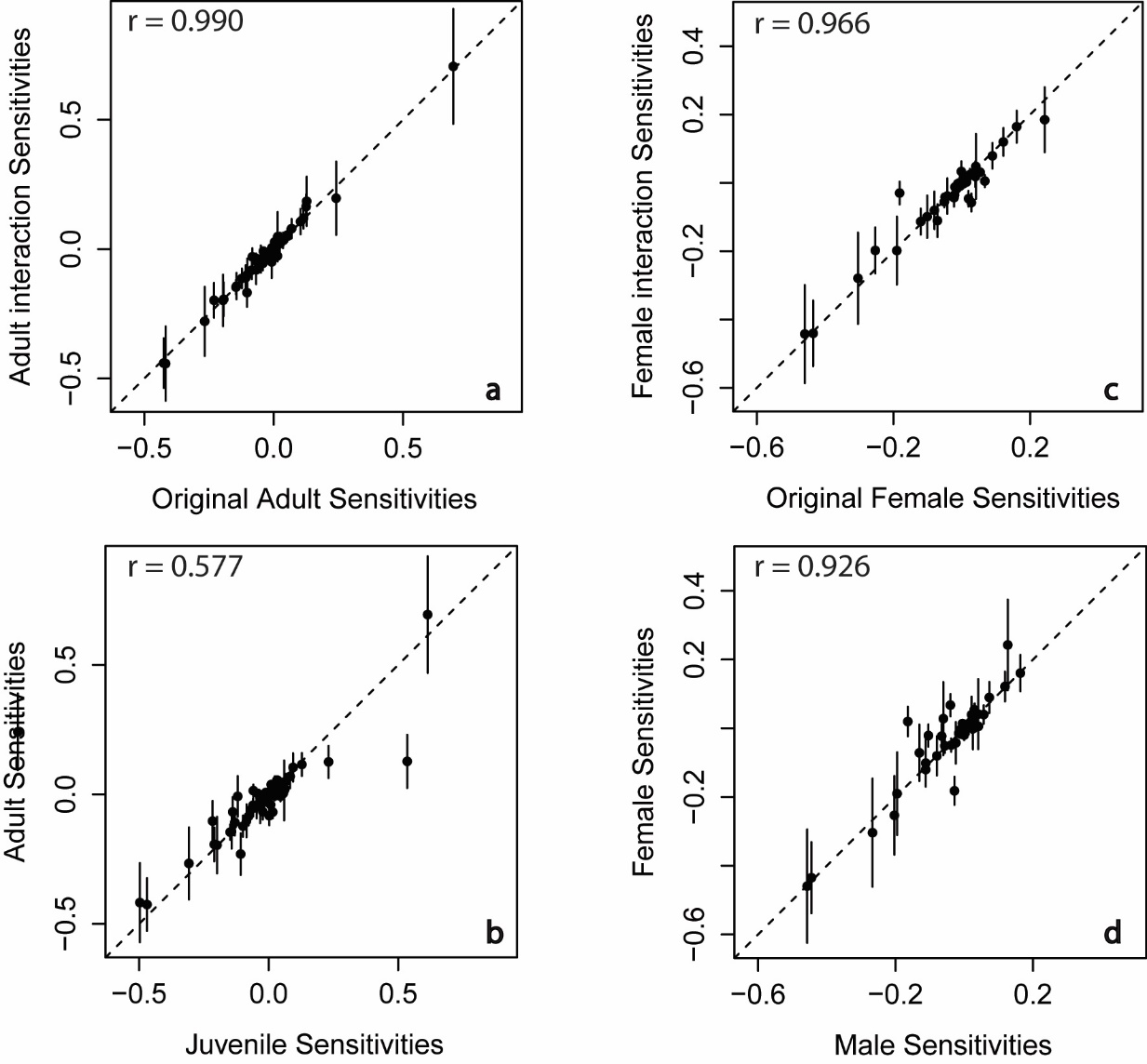


Fig E. Correlation in sensitivities between groups. Shows the correlations between (a) the original sensitivities (i.e. sensitivities without an interaction between age and climate) and adult interaction sensitivities (i.e. sensitivity values for adults with an interaction between age and climate), (b) between juvenile interaction sensitivities and adult interaction sensitivities (i.e. sensitivity values for juvenile and adults, respectively, with an interaction between age and climate), (c) the original and female sensitivities and (d) female and male sensitivities.

2.5 Climate Sensitivities

Our measure of sensitivity was the tangent at the mean climate (i.e. the first derivative of the climate regression function or the local slope estimate at the mean value of the climate variable of interest).


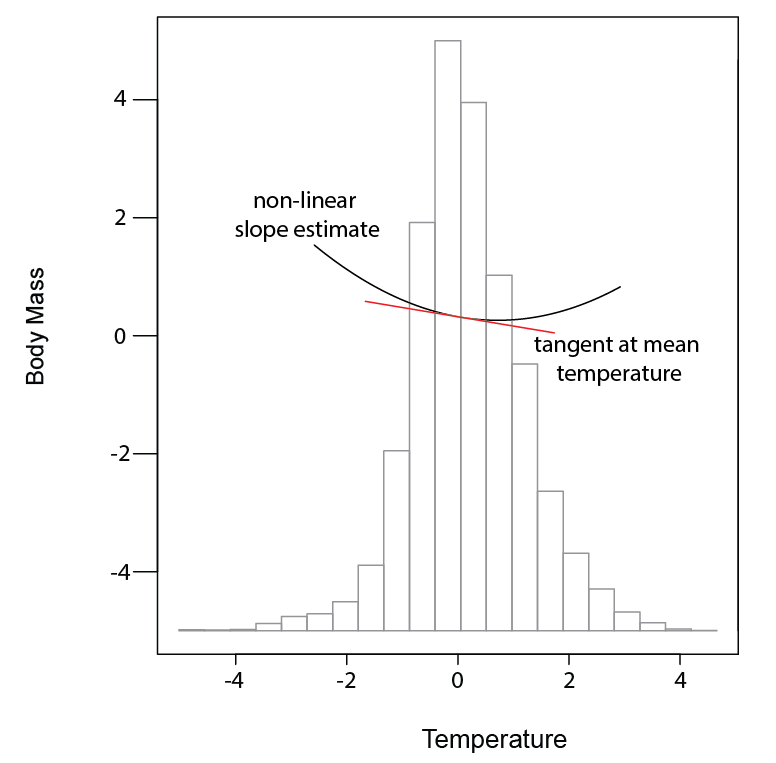


Fig F. Illustration of how the sensitivity estimate is calculated for a non-linear response between body condition and temperature. The black slope shows the non-linear relationship for a species, and the red linear slope shows the sensitivity estimate, i.e. the tangent at the mean. The histogram shows the distribution of mean annual temperature values (i.e. the range of temperatures experienced over the study period) mean centred such that 0 is the mean temperature.

2.6 Climate Vulnerabilities

We calculated seasonal projections (Winter (December-February), Spring (March-May), Summer (June-August) and Autumn (September-November)) based on Royal Netherlands Meteorological Institute’s predictions. There currently are no predictions for how the levels of sunshine are expected to change in the future under climate change scenarios.

2.7 Species and site traits

We generated matrices of phylogenetic distances between species (distances are directly proportional to time [millions of years ago]) for each tree so that our results were independent of the tree used (Fig G). Dissimilarity in climate sensitivity was calculated as the absolute difference in the climate response estimates between two species. We calculated pairwise dissimilarities and their standard errors by generating 1,000 data points centred on the sensitivity value with a distribution equal to its standard error (i.e. bootstrapping). We then calculated the differences between all 1,000 data points, with the final dissimilarity value between two sites calculated as the mean, and the standard error derived from the standard deviation of the differences.


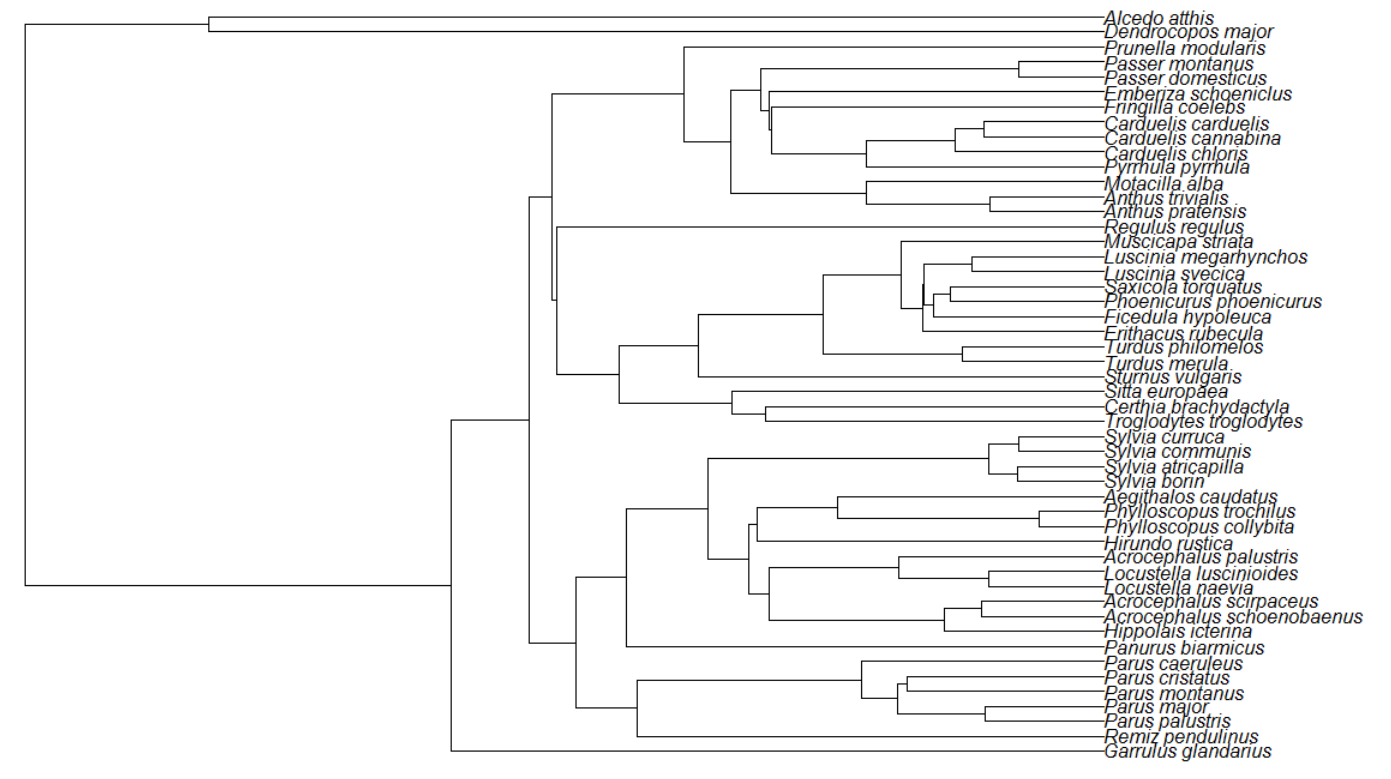


Fig G. Phylogenetic tree (from one of the 1000 trees) showing relatedness among species.

For the analysis, the pairwise dissimilarities in sensitivity estimates among each of the species were squared to improve adherence to the assumption of a normal distribution and to stop the estimates from being negative numbers (the values of dissimilarity cannot fall below 0). We used the ‘lmer()’ function from the R-package ‘lme4’ (Bates *et al.*, 2015). Both species identities in each pairwise dissimilarity value were included as a random effect in the model to account for the non-independence among values (i.e. as species 1 is compared to species 2 and 3 etc. these values are non-independent).

For the second analysis investigating whether more closely related species are impacted by the same climate variables, we carried out logistic regression using the ‘glmer’ function from the R package ‘lme4’. Dissimilarity in climate signal was scored by comparing whether two species were both affected (or both unaffected) by the same climate variable (same=0, different=1).

The analysis for spatial distances was carried out similarly to the phylogenetic distances. The two site identities of the pairwise comparison were included as random effects and the dissimilarities were squared to improve adherence to the assumption of normality. We used the ‘lmer()’ function from the R-package ‘lme4’ (Bates *et al.*, 2015).

**References**

Arnold, T. W. (2010) ‘Uninformative Parameters and Model Selection Using Akaike’s Information Criterion’, *The Journal of Wildlife Management*, 74(6), pp. 1175–1178. doi: 10.1111/j.1937-2817.2010.tb01236.x.

Bates, D. *et al.* (2015) ‘Fitting linear mixed-effects models using lme4. J. Stat. Softw. 67:1–48.’

Burnham, K. and Anderson, D. (2002) *Model selection and multimodel inference*. 2nd edn. New York: Springer.

Burnham, K. P. and Anderson, D. R. (2002) *Model selection and multi-model inference: a practical information-theoretic approach*. New York: Springer.

Freckleton, R. P. (2010) ‘Dealing with collinearity in behavioural and ecological data: model averaging and the problems of measurement error’, *Behavioral Ecology and Sociobiology*, 65(1), pp. 91–101. doi: 10.1007/s00265-010-1045-6.

Mullarney, K. *et al.* (2000) *The Complete Guide to the Birds of Europe*. London: Harper Collins.

van de Pol, M. *et al.* (2016) ‘Identifying the best climatic predictors in ecology and evolution’, *Methods in Ecology and Evolution*, 7(10), pp. 1246–1257. doi: 10.1111/2041-210X.12590.
