# Supplementary material for: High intra-specific variation in avian body condition responses to climate limits generalisation across species
Source: PLoS One. 2018 Feb 21;13(2):e0192401. doi: 10.1371/journal.pone.0192401 (PMC5821336; doi:10.1371/journal.pone.0192401)
Supplement: S2 Appendix — (DOCX) [file pone.0192401.s002.docx]

**Appendix S2: Results**

1. Climate windows and signals 1

2. Climate Sensitivities 7

3. Species Signal 10

4. Species and site traits 14

1. Climate windows and signals

Table A. Importance values, calculated as the sum of ‘Akaike weights’ over all models, for each climate variable in the final model for each species. Variables are represented by NA if they did not have any windows below the cut-off value or were subsequently removed as they appeared to be hitchhiker values. The sexed column shows which species were able to be accurately sexed, and therefore which species had sex differences included in the models. Temp is temperature, DTR is the climate variable daily temperature range.

|  | **Common name** | **Species name** | **Temp** | **Rain** | **Sun** | **Humid** | **Wind** | **DTR** | **Sexed** |
| --- | --- | --- | --- | --- | --- | --- | --- | --- | --- |
| 1 | Bearded reedling | *Panurus biarmicus* | **0.55** | **0.57** | NA | **1.00** | **0.92** | **0.92** | yes |
| 2 | Bluethroat | *Luscinia svecica* | NA | **1.00** | **0.92** | **0.64** | **0.98** | **1.00** | yes |
| 3 | Common blackbird | *Turdus merula* | **0.87** | NA | **0.57** | **0.61** | **1.00** | **1.00** | yes |
| 4 | Common chaffinch | *Fringilla coelebs* | **0.68** | **0.99** | NA | **0.65** | NA | NA | yes |
| 5 | Common chiffchaff | *Phylloscopus collybita* | **0.56** | **1.00** | **0.73** | **0.91** | **1.00** | **1.00** | no |
| 6 | Common grasshopper warbler | *Locustella naevia* | NA | NA | **0.98** | NA | **0.96** | **0.84** | no |
| 7 | Common linnet | *Carduelis cannabina* | **0.56** | **1.00** | NA | NA | **1.00** | **0.46** | yes |
| 8 | Common redstart | *Phoenicurus phoenicurus* | **0.81** | NA | **0.84** | **0.77** | NA | NA | yes |
| 9 | Common reed bunting | *Emberiza schoeniclus* | **1.00** | **0.97** | **0.98** | **1.00** | **1.00** | **0.51** | yes |
| 10 | Common starling | *Sturnus vulgaris* | **0.94** | **0.99** | **1.00** | **0.93** | **0.92** | NA | yes |
| 11 | Common whitethroat | *Sylvia communis* | NA | **0.81** | **0.97** | **0.99** | NA | 0.75 | yes |
| 12 | Dunnock | *Prunella modularis* | **0.85** | **0.94** | NA | **0.63** | **0.91** | NA | no |
| 13 | Eurasian blackcap | *Sylvia atricapilla* | **1.00** | **0.92** | NA | **0.89** | **0.89** | NA | yes |
| 14 | Eurasian blue tit | *Cyanistes caeruleus formerly Parus caeruleus* | NA | **0.99** | **0.89** | **0.78** | **1.00** | **1.00** | no |
| 15 | Eurasian bullfinch | *Pyrrhula pyrrhula* | **1.00** | NA | NA | **0.99** | NA | **0.63** | yes |
| 16 | Eurasian jay | *Garrulus glandarius* | NA | **0.98** | NA | **0.95** | **0.90** | NA | no |
| 17 | Eurasian reed warbler | *Acrocephalus scirpaceus* | **1.00** | **0.99** | **1.00** | **1.00** | NA | **1.00** | no |
| 18 | Eurasian tree sparrow | *Passer montanus* | **0.58** | NA | NA | NA | **0.53** | **0.90** | no |
| 19 | Eurasian wren | *Troglodytes troglodytes* | **0.53** | **0.99** | **0.84** | NA | **0.77** | **1.00** | no |
| 20 | European crested tit | *Lophophanes cristatus, previously Parus cristatus* | NA | **0.84** | NA | **0.87** | **0.99** | **0.92** | no |
| 21 | European goldfinch | *Carduelis carduelis* | NA | **1.00** | NA | **0.99** | NA | NA | yes |
| 22 | European greenfinch | *Carduelis chloris* | NA | **0.72** | NA | **0.94** | NA | **0.97** | yes |
| 23 | European pied flycatcher | *Ficedula hypoleuca* | NA | NA | NA | NA | **0.80** | **0.99** | yes |
| 24 | European robin | *Erithacus rubecula* | **0.99** | **0.57** | **0.89** | NA | **1.00** | NA | no |
| 25 | European stonechat | *Saxicola torquata rubicola* | NA | NA | **0.51** | **0.79** | **0.71** | **0.82** | yes |
| 26 | Garden warbler | *Sylvia borin* | NA | NA | NA | NA | NA | **1.00** | no |
| 27 | Great tit | *Parus major* | NA | **1.00** | **0.89** | **0.69** | **1.00** | **0.57** | yes |
| 28 | House sparrow | *Passer domesticus* | 0.89 | NA | NA | NA | **0.92** | **0.96** | yes |
| 29 | Icterine warbler | *Hippolais icterina* | NA | NA | NA | **1.00** | NA | NA | no |
| 30 | Long-tailed tit | *Aegithalos caudatus* | **0.99** | **0.93** | **0.58** | NA | **1.00** | NA | no |
| 31 | Marsh tit | *Poecile palustris* | NA | NA | **1.00** | NA | NA | NA | no |
| 32 | Marsh warbler | *Acrocephalus palustris* | NA | NA | **0.98** | **0.97** | **0.99** | NA | no |
| 33 | Sedge warbler | *Acrocephalus schoenobaenus* | NA | **1.00** | **1.00** | NA | **0.89** | **1.00** | no |
| 34 | Short-toed treecreeper | *Certhia brachydactyla* | 0.98 | **1.00** | **0.74** | **0.55** | NA | NA | no |
| 35 | Song thrush | *Turdus philomelos* | NA | **0.98** | **0.78** | NA | NA | **0.99** | no |
| 36 | Spotted flycatcher | *Muscicapa striata* | NA | **1.00** | NA | NA | NA | NA | no |
| 37 | Tree pipit | *Anthus trivialis* | NA | **0.97** | NA | NA | **0.77** | NA | no |
| 38 | Willow tit | *Parus montanus* | NA | NA | NA | NA | NA | **1.00** | no |
| 39 | Willow warbler | *Phylloscopus trochilus* | **1.00** | **1.00** | **0.64** | **0.78** | **1.00** | **1.00** | no |
| 40 | Common nightingale | *Luscinia megarhynchos* | NA | NA | NA | NA | NA | NA | no |
| 41 | Eurasian nuthatch | *Sitta europaea* | NA | NA | NA | NA | NA | NA | yes |
| 42 | Goldcrest | *Regulus regulus* | NA | NA | NA | NA | NA | NA | yes |
| 43 | Great spotted woodpecker | *Dendrocopos major* | NA | NA | NA | NA | NA | NA | yes |
| 44 | Lesser whitethroat | *Sylvia curruca* | NA | NA | NA | NA | NA | NA | yes |
| 45 | Savi's warbler | *Locustella luscinioides* | NA | NA | NA | NA | NA | NA | no |
| 46 | White wagtail | *Motacilla alba* | NA | NA | NA | NA | NA | NA | yes |


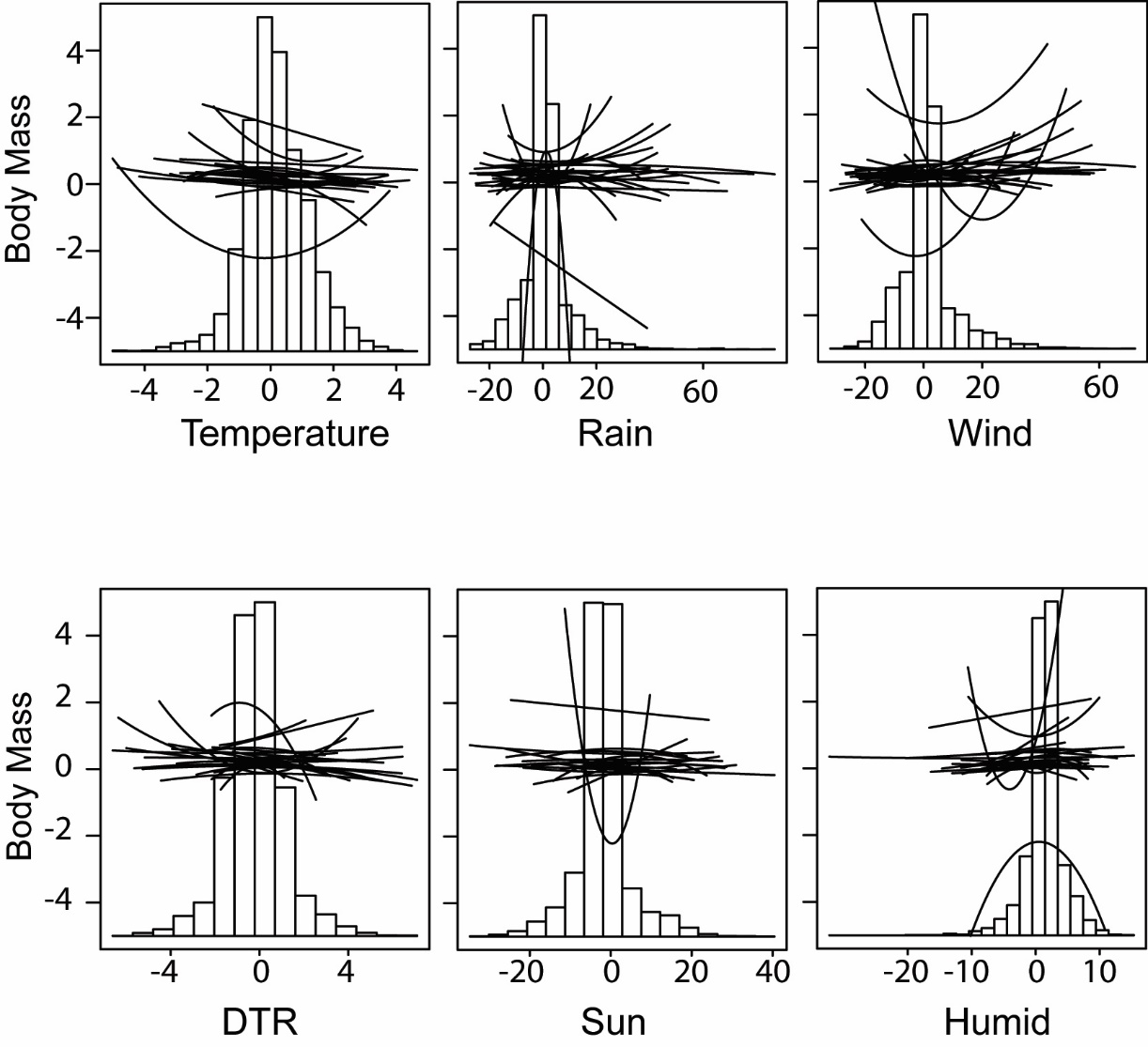


Fig A. Model averaged slope estimates for the climate impacts on body condition. Each line shows the relationship between climate and body condition for a different species. The histogram shows the distribution of mean annual climate values (i.e. the range of climate experienced over the study period).

Table B. Climate windows for all species. Blank spaces indicate where those climate variables had no effect on body condition. From and till indicate the earliest and latest dates of the windows, respectively. Italicised dates indicate secondary windows. Temp is temperature and CTR is daily temperature range. The I.D. refers to the Species I.D. in Fig 3.

|  |  | **Temp** |  | **Rain** |  | **Sun** |  | **Humid** |  | **DTR** |  | **Wind** |  |
| --- | --- | --- | --- | --- | --- | --- | --- | --- | --- | --- | --- | --- | --- |
| **I.D.** | **Species** | **from** | **till** | **from** | **till** | **from** | **till** | **from** | **till** | **from** | **till** | **from** | **till** |
| 1 | Bearded reedling | 1-Sep | 17-Oct | 23-Jun | 1-Jul |  |  | 20-Nov | 13-Feb | 6-Aug | 11-Aug | 2-Apr | 8-Apr |
| 2 | Bluethroat |  |  | 2-May | 7-May | 6-Nov | 14-Dec | 15-Jan | 30-Jan | 19-May | 24-May | 2-Apr | 7-Apr |
| 3 | Common blackbird | 29-May | 12-Jul |  |  | 24-Jun | 18-Jul | 27-Jun | 17-Jul | 2-Jan | 30-Jan | 20-Nov | 24-Dec |
| 4 | Common chaffinch | 29-May | 23-Jul | 20-Apr | 30-Apr |  |  | 27-Jun | 9-Aug |  |  |  |  |
| 5 | Common chiffchaff | 25-Jul | 6-Aug | 3-Jun | 8-Jun | 12-May | 17-May | 12-Mar | 10-Aug | 5-Jul  *4-Feb* | 2-Aug  *17-Feb* | 8-Feb | 18-Feb |
| 6 | Common grasshopper warbler |  |  |  |  | 30-May | 9-Jun |  |  | 7-Apr | 7-Jul | 21-Sep | 11-Oct |
| 7 | Common linnet | 29-May | 9-Jul | 13-Dec | 28-Dec |  |  |  |  | 23-Mar | 29-Apr | 18-Feb | 27-Feb |
| 8 | Common redstart | 13-Jun | 1-Jul |  |  | 14-Feb | 22-Apr | 9-Jul | 14-Jul |  |  |  |  |
| 9 | Common reed bunting | 29-May | 15-Jun | 20-Apr | 25-Apr | 27-Dec | 20-Jan | 30-Oct | 26-Apr | 12-Sep | 5-Jul | 26-Aug | 21-Nov |
| 10 | Common starling | 5-Mar | 12-Mar | 4-Dec | 27-Dec | 14-Jan | 14-Mar | 4-Mar | 9-Mar |  |  | 24-Nov | 5-Feb |
| 11 | Common whitethroat |  |  | 10-Feb | 17-Feb | 2-Feb | 22-Feb | 9-May | 30-Jun | 5-Jan | 25-Jan |  |  |
| 12 | Dunnock | 28-May | 2-Jul | 11-Sep | 14-Oct |  |  | 6-Apr | 22-May |  |  | 9-Feb | 25-Feb |
| 13 | Eurasian blackcap | 12-May | 9-Jul | 24-Jun | 2-Jul |  |  | 25-Jun | 2-Jul |  |  | 4-Apr | 9-Apr |
| 14 | Eurasian blue tit |  |  | 24-Nov | 25-Jun | 7-Jul | 19-Jul | 19-Jun  *24-Dec* | 3-Aug  *21-Jan* | 10-Jan | 26-Jan | 12-Jun  *22-Jan* | 20-Jun  *20-Feb* |
| 15 | Eurasian bullfinch | 8-Jun | 29-Jun |  |  |  |  | 20-Jan | 4-Feb | 4-Dec | 19-Dec |  |  |
| 16 | Eurasian jay |  |  | 14-Mar | 3-Jul |  |  | 11-Sep | 28-Sep |  |  | 2-Mar | 7-Mar |
| 17 | Eurasian reed warbler | 14-May | 23-May | 8-Nov | 1-Dec | 3-Apr | 25-May | 18-Jan | 13-Feb | 21-Jan  *16-May* | 6-Feb  *21-May* |  |  |
| 18 | Eurasian tree sparrow | 22-Sep | 18-May |  |  |  |  |  |  | 1-Feb | 19-Feb | 21-Feb | 27-Feb |
| 19 | Eurasian wren | 28-May | 8-Jul | 9-Sep  *16-Feb* | 6-Oct  *23-Feb* | 2-Feb | 1-Mar |  |  | 11-Apr | 21-Apr | 11-Feb | 22-Feb |
| 20 | European crested tit |  |  | 8-Feb | 17-Feb |  |  | 29-Aug | 17-Sep | 21-Nov | 21-Dec | 11-Mar | 2-May |
| 21 | European goldfinch |  |  | 30-Apr | 12-May |  |  | 18-Dec | 2-Jan |  |  |  |  |
| 22 | European greenfinch |  |  | 17-Sep | 28-Nov |  |  | 22-Aug | 24-Jul | 12-Feb | 17-Feb |  |  |
| 23 | European pied flycatcher |  |  |  |  |  |  |  |  | 30-May | 16-Jun | 8-Mar | 27-Mar |
| 24 | European robin | 28-May | 8-Jul | 26-Aug | 30-Sep | 28-Jan | 25-Feb |  |  |  |  | 27-Jan  *28-Mar* | 7-Mar  *2-Apr* |
| 25 | European stonechat |  |  |  |  | 18-Nov | 12-Dec | 6-Oct | 13-Nov | 24-Dec | 9-Feb | 2-Sep | 28-Sep |
| 26 | Garden warbler |  |  |  |  |  |  |  |  | 8-Feb | 17-Feb |  |  |
| 27 | Great tit |  |  | 7-Dec | 17-Apr | 5-Jun | 17-Jun | 27-Feb | 7-Mar | 6-Jun | 17-Jun | 16-Feb | 21-Feb |
| 28 | House sparrow | 14-May  *12-Mar* | 4-Jun  *26-Mar* |  |  |  |  |  |  | 22-May | 31-May | 11-Feb | 23-Feb |
| 29 | Icterine warbler |  |  |  |  |  |  | 15-Apr | 15-Aug |  |  |  |  |
| 30 | Long-tailed tit | 9-Jun | 22-Jun | 2-Feb  *7-Aug* | 25-Feb  *13-Aug* | 7-Jul | 14-Jul |  |  |  |  | 3-Apr | 11-Apr |
| 31 | Marsh tit |  |  |  |  | 10-May | 15-Aug |  |  |  |  |  |  |
| 32 | Marsh warbler |  |  |  |  | 17-Aug | 19-Jul | 20-Jun | 19-Jul |  |  | 5-May | 10-May |
| 33 | Sedge warbler |  |  | 27-Aug | 18-Oct | 16-Aug | 7-Mar |  |  | 21-Nov | 8-Dec | 18-May | 23-May |
| 34 | Short-toed treecreeper | 27-Apr | 17-May | 20-May | 6-Jun | 25-Jul | 31-Jul | 24-Jul | 12-Aug |  |  |  |  |
| 35 | Song thrush |  |  | 15-Nov | 26-Jan | 19-Oct | 8-Dec |  |  | 2-Mar | 7-Mar |  |  |
| 36 | Spotted flycatcher |  |  | 7-Dec | 23-Dec |  |  |  |  |  |  |  |  |
| 37 | Tree pipit |  |  | 28-Aug | 15-Sep |  |  |  |  |  |  | 3-Feb | 24-Feb |
| 38 | Willow tit |  |  |  |  |  |  |  |  | 18-Nov | 18-Feb |  |  |
| 39 | Willow warbler | 10-Jun | 27-Jun | 28-Aug  *11-Nov* | 12-Sep  *11-Dec* | 25-Jan | 11-Mar | 17-Mar | 22-Mar | 19-May | 25-May | 27-Aug  *20-May* | 11-Sep  *25-May* |

Climate Sensitivities

Table C. The sensitivity estimates (as % change in total body condition per climate unit) for each species and climate variable. The % column gives the sensitivity, the SE column shows the standard error, and the Lin column describes whether the relationship between mass and each climate variable was linear (L), quadratic (Q), or no relationship was found (NA).

|  |  |  | **Temperature** | | | **Humid** | | | **Rain** | | | **Wind** | | | **DTR** | | | **Sunshine** | | |
| --- | --- | --- | --- | --- | --- | --- | --- | --- | --- | --- | --- | --- | --- | --- | --- | --- | --- | --- | --- | --- |
|  | **Common name** | **Species name** | % | SE | Lin | % | SE | Lin | % | SE | Lin | % | SE | Lin | % | SE | Lin | % | SE | Lin |
| 1 | Bearded reedling | *Panurus biarmicus* | 0.559 | 0.266 | Q | -0.057 | 0.103 | Q | -0.013 | 0.008 | L | -0.019 | 0.022 | Q | 0.056 | 0.069 | Q | NA | NA | NA |
| 2 | Bluethroat | *Luscinia svecica* | NA | NA | NA | -0.057 | 0.032 | L | -0.038 | 0.008 | Q | -0.019 | 0.011 | Q | -0.105 | 0.044 | Q | -0.031 | 0.023 | Q |
| 3 | Common blackbird | *Turdus merula* | -0.308 | 0.147 | L | 0.038 | 0.031 | L | NA | NA | NA | -0.020 | 0.010 | Q | -0.488 | 0.158 | Q | -0.013 | 0.011 | L |
| 4 | Common chaffinch | *Fringilla coelebs* | -0.372 | 0.254 | L | 0.110 | 0.078 | L | 0.055 | 0.020 | Q | NA | NA | NA | NA | NA | NA | NA | NA | NA |
| 5 | Common chiffchaff | *Phylloscopus collybita* | -0.087 | 0.056 | L | 0.035 | 0.012 | Q | 0.014 | 0.004 | Q | 0.033 | 0.008 | Q | -0.180 | 0.047 | Q | 0.009 | 0.007 | Q |
| 6 | Common grasshopper warbler | *Locustella naevia* | NA | NA | NA | NA | NA | NA | NA | NA | NA | 0.049 | 0.035 | Q | -0.452 | 0.207 | Q | -0.037 | 0.016 | Q |
| 7 | Common Lnet | *Carduelis cannabina* | -0.559 | 0.350 | L | NA | NA | NA | 0.044 | 0.021 | Q | 0.061 | 0.025 | Q | -0.245 | 0.147 | L | NA | NA | NA |
| 8 | Common redstart | *Phoenicurus phoenicurus* | -0.764 | 0.332 | L | 0.171 | 0.077 | L | NA | NA | NA | NA | NA | NA | NA | NA | NA | 0.151 | 0.056 | Q |
| 9 | Common reed bunting | *Emberiza schoeniclus* | -0.234 | 0.060 | Q | 0.174 | 0.046 | L | -0.013 | 0.013 | Q | 0.013 | 0.021 | Q | -0.167 | 0.186 | Q | 0.015 | 0.013 | Q |
| 10 | Common starLg | *Sturnus vulgaris* | 0.067 | 0.134 | Q | 0.035 | 0.053 | Q | -0.077 | 0.023 | L | 0.026 | 0.027 | Q | NA | NA | NA | -0.054 | 0.073 | Q |
| 11 | Common whitethroat | *Sylvia communis* | NA | NA | NA | 0.155 | 0.049 | Q | 0.007 | 0.011 | Q | NA | NA | NA | -0.334 | 0.166 | L | -0.024 | 0.013 | Q |
| 12 | Dunnock | *Prunella modularis* | -0.437 | 0.182 | L | 0.003 | 0.042 | Q | 0.034 | 0.014 | Q | 0.026 | 0.010 | L | NA | NA | NA | NA | NA | NA |
| 13 | Eurasian blackcap | *Sylvia atricapilla* | -0.206 | 0.108 | Q | 0.057 | 0.017 | L | 0.013 | 0.005 | Q | 0.027 | 0.007 | Q | NA | NA | NA | NA | NA | NA |
| 14 | Eurasian blue tit | *Parus caeruleus* | NA | NA | NA | 0.092 | 0.030 | Q | -0.005 | 0.021 | Q | -0.015 | 0.011 | Q | -0.316 | 0.095 | Q | -0.004 | 0.007 | Q |
| 15 | Eurasian bullfinch | *Pyrrhula pyrrhula* | -1.996 | 0.435 | Q | 0.024 | 0.080 | Q | NA | NA | NA | NA | NA | NA | 0.838 | 0.432 | Q | NA | NA | NA |
| 16 | Eurasian jay | *Garrulus glandarius* | NA | NA | NA | 0.449 | 0.141 | Q | 0.125 | 0.090 | Q | -0.123 | 0.041 | Q | NA | NA | NA | NA | NA | NA |
| 17 | Eurasian reed warbler | *Acrocephalus scirpaceus* | 0.053 | 0.029 | Q | -0.059 | 0.014 | L | -0.016 | 0.005 | Q | NA | NA | NA | 0.239 | 0.050 | Q | -0.037 | 0.008 | Q |
| 18 | Eurasian tree sparrow | *Passer montanus* | -0.818 | 0.267 | Q | NA | NA | NA | NA | NA | NA | -0.047 | 0.026 | Q | 0.518 | 0.237 | Q | NA | NA | NA |
| 19 | Eurasian wren | *Troglodytes troglodytes* | -0.284 | 0.206 | L | NA | NA | NA | 0.023 | 0.009 | Q | 0.020 | 0.009 | L | -0.250 | 0.058 | Q | 0.013 | 0.011 | Q |
| 20 | European crested tit | *Parus cristatus* | NA | NA | NA | 0.212 | 0.110 | Q | -0.015 | 0.019 | Q | -0.381 | 0.085 | Q | -1.302 | 0.464 | L | NA | NA | NA |
| 21 | European goldfinch | *Carduelis carduelis* | NA | NA | NA | 0.001 | 0.104 | Q | -0.020 | 0.019 | Q | NA | NA | NA | NA | NA | NA | NA | NA | NA |
| 22 | European greenfinch | *Carduelis chloris* | NA | NA | NA | 0.455 | 0.155 | L | -0.012 | 0.036 | Q | NA | NA | NA | 0.624 | 0.178 | L | NA | NA | NA |
| 23 | European pied flycatcher | *Ficedula hypoleuca* | NA | NA | NA | NA | NA | NA | NA | NA | NA | 0.045 | 0.047 | Q | 0.273 | 0.245 | Q | NA | NA | NA |
| 24 | European robin | *Erithacus rubecula* | -0.901 | 0.171 | L | NA | NA | NA | 0.010 | 0.013 | Q | 0.025 | 0.013 | Q | NA | NA | NA | 0.025 | 0.012 | Q |
|  | European stonechat | *Saxicola rubicola* | NA | NA | NA | -0.420 | 0.193 | L | NA | NA | NA | 0.114 | 0.055 | Q | -1.447 | 0.728 | L | 0.099 | 0.066 | Q |
| 26 | Garden warbler | *Sylvia borin* | NA | NA | NA | NA | NA | NA | NA | NA | NA | NA | NA | NA | 0.189 | 0.073 | Q | NA | NA | NA |
| 27 | Great tit | *Parus major* | NA | NA | NA | -0.014 | 0.018 | Q | -0.011 | 0.014 | Q | 0.036 | 0.006 | Q | 0.053 | 0.040 | Q | -0.013 | 0.009 | Q |
| 28 | House sparrow | *Passer domesticus* | -0.709 | 0.242 | Q | NA | NA | NA | NA | NA | NA | 0.062 | 0.020 | L | -0.406 | 0.136 | L | NA | NA | NA |
| 29 | Icterine warbler | *Hippolais icterina* | NA | NA | NA | 0.256 | 0.085 | Q | NA | NA | NA | NA | NA | NA | NA | NA | NA | NA | NA | NA |
| 30 | Long-tailed tit | *Aegithalos caudatus* | -0.280 | 0.170 | Q | NA | NA | NA | 0.060 | 0.024 | Q | 0.059 | 0.026 | Q | NA | NA | NA | -0.010 | 0.016 | Q |
| 31 | Marsh tit | *Parus palustris* | NA | NA | NA | NA | NA | NA | NA | NA | NA | NA | NA | NA | NA | NA | NA | -0.265 | 0.068 | Q |
| 32 | Marsh warbler | *Acrocephalus palustris* | NA | NA | NA | 0.084 | 0.026 | L | NA | NA | NA | 0.001 | 0.009 | Q | NA | NA | NA | -0.162 | 0.041 | L |
| 33 | Sedge warbler | *Acroce-phalus schoenobaenus* | NA | NA | NA | NA | NA | NA | 0.031 | 0.007 | Q | -0.016 | 0.006 | L | 0.423 | 0.081 | L | 0.033 | 0.031 | Q |
| 34 | Short-toed treecreeper | *Certhia brachydactyla* | -0.398 | 0.154 | Q | 0.140 | 0.102 | L | -0.017 | 0.028 | Q | NA | NA | NA | NA | NA | NA | -0.048 | 0.027 | L |
| 35 | Song thrush | *Turdus philomelos* | NA | NA | NA | NA | NA | NA | -0.035 | 0.029 | Q | NA | NA | NA | -0.072 | 0.091 | Q | 0.079 | 0.037 | L |
| 36 | Spotted flycatcher | *Muscicapa striata* | NA | NA | NA | NA | NA | NA | 0.029 | 0.040 | Q | NA | NA | NA | NA | NA | NA | NA | NA | NA |
| 37 | Tree pipit | *Anthus trivialis* | NA | NA | NA | NA | NA | NA | 0.242 | 0.050 | Q | 0.164 | 0.053 | L | NA | NA | NA | NA | NA | NA |
| 38 | Willow tit | *Parus montanus* | NA | NA | NA | NA | NA | NA | NA | NA | NA | NA | NA | NA | -0.430 | 0.401 | Q | NA | NA | NA |
| 39 | Willow warbler | *Phylloscopus trochilus* | -0.342 | 0.065 | Q | -0.038 | 0.018 | L | 0.030 | 0.005 | Q | 0.008 | 0.006 | Q | -0.068 | 0.026 | Q | 0.011 | 0.012 | Q |


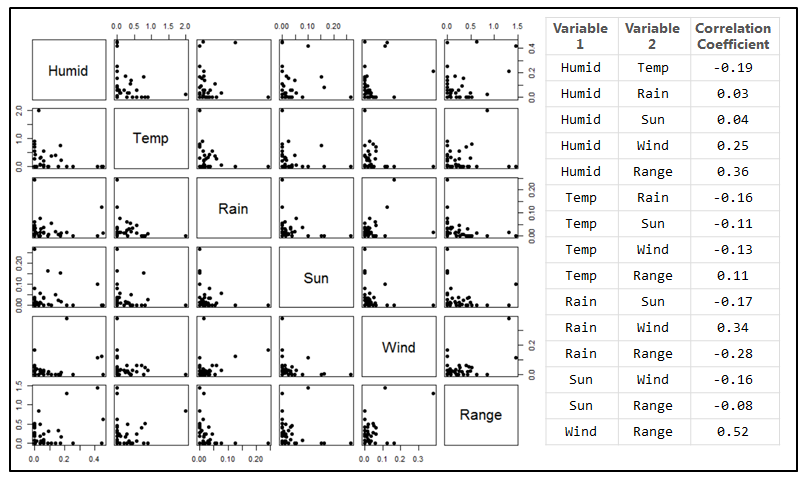


Fig B. Correlations between species sensitivities to climate variables. Temp is temperature and range is daily temperature range.

Species Signal

Table D. The amount of variance explained by the random factor species ($\boldsymbol{u}$), the within-species variance ($\boldsymbol{v}$) (including sampling variance), and the % among-species variation. The % among-species variation is calculated as $\frac{\boldsymbol{u}}{\boldsymbol{(u+v)}}$. DTR is daily temperature range.

| **Climate Variables** | **u** | **V** | **% among-species variation** |
| --- | --- | --- | --- |
| Temperature | 0.03025 | 1.840 | 1.6 |
| Wind | 0.00046 | 0.286 | 0.2 |
| Sun | 0.00007 | 0.204 | 0.03 |
| Rain | 0.00001 | 0.210 | 0.005 |
| Humid | 0.00000 | 0.415 | 0.000 |
| DTR | 0.00000 | 2.289 | 0.000 |
| Vulnerability | 0.00000 | 2.722 | 0.000 |

Table E. Model selection table for all combinations of species characteristics explaining variation in climate sensitivities. Shows the best to worst models for each of the six climate variables (ranked by AICc). Each model included either all or none of the habitat characteristics (including open, urban, wet and woodland habitats). The column Int. shows the intercept value, df the degrees of freedom and logLik the log-likelihood value. DTR is the daily temperature range.

| **Climate variable** | **Int.** | **Body Cond.** | **Migrat-ion** | **Life-span** | **Open** | **Urban** | **Wet** | **Wood-land** | **R^2^** | **df** | **logLik** | **AICc** |
| --- | --- | --- | --- | --- | --- | --- | --- | --- | --- | --- | --- | --- |
| Humid | 0.040 | NA | NA | NA | NA | NA | NA | NA | 0.000 | 2.000 | 16.436 | -28.301 |
| Humid | -0.122 | NA | NA | 0.096 | NA | NA | NA | NA | 0.080 | 3.000 | 17.437 | -27.675 |
| Humid | 0.015 | 0.001 | NA | NA | NA | NA | NA | NA | 0.078 | 3.000 | 17.414 | -27.628 |
| Humid | 0.072 | NA | -0.028 | NA | NA | NA | NA | NA | 0.060 | 3.000 | 17.173 | -27.146 |
| Humid | -0.106 | 0.001 | NA | 0.075 | NA | NA | NA | NA | 0.124 | 4.000 | 18.019 | -25.934 |
| Humid | -0.076 | NA | -0.023 | 0.084 | NA | NA | NA | NA | 0.119 | 4.000 | 17.959 | -25.812 |
| Humid | 0.042 | 0.001 | -0.018 | NA | NA | NA | NA | NA | 0.100 | 4.000 | 17.701 | -25.296 |
| Humid | 0.058 | NA | NA | NA | -0.115 | 0.031 | -0.078 | -0.005 | 0.263 | 6.000 | 20.098 | -23.255 |
| Humid | -0.077 | 0.001 | -0.016 | 0.072 | NA | NA | NA | NA | 0.141 | 5.000 | 18.260 | -23.187 |
| Humid | -0.106 | NA | NA | 0.104 | -0.113 | 0.023 | -0.100 | -0.009 | 0.352 | 7.000 | 21.642 | -22.284 |
| Humid | 0.056 | 0.001 | NA | NA | -0.116 | 0.018 | -0.084 | -0.011 | 0.286 | 7.000 | 20.472 | -19.944 |
| Humid | 0.087 | NA | -0.013 | NA | -0.122 | 0.018 | -0.086 | -0.014 | 0.271 | 7.000 | 20.236 | -19.472 |
| Humid | -0.096 | 0.000 | NA | 0.097 | -0.113 | 0.017 | -0.101 | -0.011 | 0.357 | 8.000 | 21.728 | -17.856 |
| Humid | -0.090 | NA | -0.005 | 0.101 | -0.115 | 0.018 | -0.102 | -0.013 | 0.353 | 8.000 | 21.668 | -17.735 |
| Humid | 0.075 | 0.001 | -0.008 | NA | -0.120 | 0.010 | -0.089 | -0.016 | 0.289 | 8.000 | 20.531 | -15.462 |
| Humid | -0.085 | 0.000 | -0.004 | 0.096 | -0.115 | 0.014 | -0.103 | -0.014 | 0.357 | 9.000 | 21.740 | -12.622 |
| Temp | -0.247 | NA | NA | NA | NA | NA | NA | NA | 0.000 | 2.000 | -9.851 | 24.452 |
| Temp | -0.388 | NA | 0.136 | NA | NA | NA | NA | NA | 0.124 | 3.000 | -8.598 | 24.796 |
| Temp | 0.188 | NA | NA | -0.262 | NA | NA | NA | NA | 0.040 | 3.000 | -9.465 | 26.529 |
| Temp | -0.241 | 0.000 | NA | NA | NA | NA | NA | NA | 0.000 | 3.000 | -9.848 | 27.296 |
| Temp | -0.069 | NA | 0.126 | -0.186 | NA | NA | NA | NA | 0.143 | 4.000 | -8.385 | 27.627 |
| Temp | -0.441 | 0.002 | 0.150 | NA | NA | NA | NA | NA | 0.135 | 4.000 | -8.477 | 27.811 |
| Temp | 0.285 | -0.002 | NA | -0.302 | NA | NA | NA | NA | 0.047 | 4.000 | -9.390 | 29.636 |
| Temp | -0.153 | 0.001 | 0.136 | -0.154 | NA | NA | NA | NA | 0.146 | 5.000 | -8.354 | 31.324 |
| Temp | -0.443 | NA | NA | NA | 0.079 | -0.184 | 0.425 | 0.173 | 0.267 | 6.000 | -6.897 | 32.794 |
| Temp | 0.474 | NA | NA | -0.533 | -0.003 | -0.193 | 0.457 | 0.114 | 0.410 | 7.000 | -4.834 | 33.850 |
| Temp | -0.607 | NA | 0.107 | NA | 0.055 | -0.116 | 0.453 | 0.221 | 0.330 | 7.000 | -6.045 | 36.272 |
| Temp | -0.523 | 0.005 | NA | NA | 0.132 | -0.328 | 0.437 | 0.188 | 0.326 | 7.000 | -6.106 | 36.393 |
| Temp | 0.264 | NA | 0.068 | -0.471 | -0.009 | -0.149 | 0.471 | 0.151 | 0.433 | 8.000 | -4.452 | 39.305 |
| Temp | 0.327 | 0.003 | NA | -0.471 | 0.033 | -0.265 | 0.460 | 0.128 | 0.424 | 8.000 | -4.615 | 39.631 |
| Temp | -0.721 | 0.006 | 0.122 | NA | 0.112 | -0.271 | 0.470 | 0.245 | 0.406 | 8.000 | -4.908 | 40.216 |
| Temp | 0.002 | 0.004 | 0.086 | -0.369 | 0.041 | -0.239 | 0.478 | 0.181 | 0.458 | 9.000 | -4.024 | 46.048 |
| DTR | -0.047 | NA | NA | NA | NA | NA | NA | NA | 0.000 | 2.000 | -9.162 | 22.895 |
| DTR | -0.097 | NA | 0.046 | NA | NA | NA | NA | NA | 0.022 | 3.000 | -8.892 | 24.985 |
| DTR | -0.011 | -0.002 | NA | NA | NA | NA | NA | NA | 0.013 | 3.000 | -9.008 | 25.215 |
| DTR | -0.119 | NA | NA | 0.043 | NA | NA | NA | NA | 0.001 | 3.000 | -9.152 | 25.503 |
| DTR | -0.068 | -0.001 | 0.038 | NA | NA | NA | NA | NA | 0.026 | 4.000 | -8.850 | 27.806 |
| DTR | -0.118 | NA | 0.045 | 0.013 | NA | NA | NA | NA | 0.022 | 4.000 | -8.891 | 27.888 |
| DTR | -0.124 | -0.002 | NA | 0.070 | NA | NA | NA | NA | 0.015 | 4.000 | -8.981 | 28.068 |
| DTR | -0.121 | -0.001 | 0.036 | 0.034 | NA | NA | NA | NA | 0.026 | 5.000 | -8.844 | 31.022 |
| DTR | -0.196 | NA | NA | NA | 0.079 | 0.178 | 0.228 | -0.010 | 0.094 | 6.000 | -7.981 | 32.903 |
| DTR | -0.167 | -0.004 | NA | NA | 0.087 | 0.252 | 0.249 | 0.004 | 0.135 | 7.000 | -7.423 | 35.846 |
| DTR | -0.220 | NA | 0.073 | NA | -0.041 | 0.209 | 0.155 | -0.058 | 0.131 | 7.000 | -7.472 | 35.944 |
| DTR | 0.047 | NA | NA | -0.178 | 0.115 | 0.218 | 0.280 | 0.018 | 0.105 | 7.000 | -7.834 | 36.668 |
| DTR | 0.180 | NA | 0.093 | -0.299 | -0.013 | 0.284 | 0.223 | -0.023 | 0.160 | 8.000 | -7.073 | 39.745 |
| DTR | -0.191 | -0.003 | 0.058 | NA | -0.009 | 0.262 | 0.187 | -0.036 | 0.157 | 8.000 | -7.117 | 39.833 |
| DTR | 0.095 | -0.004 | NA | -0.192 | 0.126 | 0.296 | 0.305 | 0.034 | 0.148 | 8.000 | -7.244 | 40.088 |
| DTR | 0.195 | -0.003 | 0.077 | -0.289 | 0.017 | 0.333 | 0.252 | -0.003 | 0.183 | 9.000 | -6.733 | 44.323 |
| Sunshine | -0.007 | NA | NA | NA | NA | NA | NA | NA | 0.000 | 2.000 | 31.992 | -59.318 |
| Sunshine | 0.071 | NA | NA | -0.047 | NA | NA | NA | NA | 0.041 | 3.000 | 32.429 | -57.446 |
| Sunshine | -0.005 | NA | -0.003 | NA | NA | NA | NA | NA | 0.003 | 3.000 | 32.024 | -56.636 |
| Sunshine | -0.009 | 0.000 | NA | NA | NA | NA | NA | NA | 0.002 | 3.000 | 32.014 | -56.616 |
| Sunshine | 0.082 | NA | -0.005 | -0.051 | NA | NA | NA | NA | 0.049 | 4.000 | 32.522 | -54.544 |
| Sunshine | 0.069 | 0.000 | NA | -0.048 | NA | NA | NA | NA | 0.044 | 4.000 | 32.460 | -54.420 |
| Sunshine | -0.007 | 0.000 | -0.002 | NA | NA | NA | NA | NA | 0.004 | 4.000 | 32.033 | -53.565 |
| Sunshine | 0.080 | 0.000 | -0.004 | -0.051 | NA | NA | NA | NA | 0.050 | 5.000 | 32.529 | -51.058 |
| Sunshine | -0.043 | NA | NA | NA | 0.067 | -0.020 | 0.019 | 0.048 | 0.133 | 6.000 | 33.490 | -48.980 |
| Sunshine | 0.029 | NA | NA | -0.049 | 0.076 | -0.017 | 0.031 | 0.054 | 0.173 | 7.000 | 33.981 | -45.347 |
| Sunshine | -0.043 | 0.000 | NA | NA | 0.064 | -0.027 | 0.015 | 0.045 | 0.149 | 7.000 | 33.690 | -44.765 |
| Sunshine | -0.036 | NA | -0.006 | NA | 0.071 | -0.023 | 0.021 | 0.047 | 0.143 | 7.000 | 33.611 | -44.606 |
| Sunshine | 0.058 | NA | -0.010 | -0.061 | 0.085 | -0.021 | 0.038 | 0.053 | 0.200 | 8.000 | 34.330 | -40.661 |
| Sunshine | 0.030 | 0.000 | NA | -0.050 | 0.074 | -0.024 | 0.027 | 0.051 | 0.190 | 8.000 | 34.207 | -40.414 |
| Sunshine | -0.038 | 0.000 | -0.004 | NA | 0.067 | -0.028 | 0.017 | 0.044 | 0.155 | 8.000 | 33.760 | -39.521 |
| Sunshine | 0.055 | 0.000 | -0.008 | -0.060 | 0.082 | -0.026 | 0.034 | 0.051 | 0.210 | 9.000 | 34.466 | -34.569 |
| Wind | 0.011 | NA | NA | NA | NA | NA | NA | NA | 0.000 | 2.000 | 37.841 | -71.137 |
| Wind | 0.021 | -0.001 | NA | NA | NA | NA | NA | NA | 0.074 | 3.000 | 38.797 | -70.452 |
| Wind | 0.057 | NA | NA | -0.028 | NA | NA | NA | NA | 0.035 | 3.000 | 38.291 | -69.438 |
| Wind | 0.008 | NA | 0.002 | NA | NA | NA | NA | NA | 0.002 | 3.000 | 37.871 | -68.599 |
| Wind | 0.041 | 0.000 | NA | -0.013 | NA | NA | NA | NA | 0.079 | 4.000 | 38.874 | -67.748 |
| Wind | 0.024 | -0.001 | -0.002 | NA | NA | NA | NA | NA | 0.075 | 4.000 | 38.820 | -67.640 |
| Wind | 0.055 | NA | 0.002 | -0.028 | NA | NA | NA | NA | 0.036 | 4.000 | 38.305 | -66.609 |
| Wind | 0.042 | 0.000 | -0.002 | -0.012 | NA | NA | NA | NA | 0.081 | 5.000 | 38.892 | -64.625 |
| Wind | 0.021 | NA | NA | NA | 0.003 | -0.002 | -0.022 | -0.007 | 0.032 | 6.000 | 38.243 | -59.819 |
| Wind | 0.025 | -0.001 | NA | NA | 0.001 | 0.009 | -0.019 | -0.002 | 0.120 | 7.000 | 39.442 | -58.297 |
| Wind | 0.062 | NA | NA | -0.027 | 0.003 | 0.001 | -0.019 | -0.006 | 0.063 | 7.000 | 38.649 | -56.711 |
| Wind | 0.013 | NA | 0.005 | NA | 0.003 | 0.001 | -0.021 | -0.004 | 0.037 | 7.000 | 38.317 | -56.046 |
| Wind | 0.039 | -0.001 | NA | -0.009 | 0.001 | 0.009 | -0.018 | -0.003 | 0.123 | 8.000 | 39.485 | -53.971 |
| Wind | 0.021 | -0.001 | 0.002 | NA | 0.001 | 0.010 | -0.019 | -0.001 | 0.122 | 8.000 | 39.461 | -53.921 |
| Wind | 0.054 | NA | 0.004 | -0.027 | 0.003 | 0.004 | -0.019 | -0.003 | 0.068 | 8.000 | 38.719 | -52.438 |
| Wind | 0.035 | -0.001 | 0.002 | -0.010 | 0.001 | 0.011 | -0.018 | -0.001 | 0.125 | 9.000 | 39.506 | -49.012 |
| Rain | 0.008 | NA | NA | NA | NA | NA | NA | NA | 0.000 | 2.000 | 46.508 | -88.495 |
| Rain | -0.031 | NA | NA | 0.024 | NA | NA | NA | NA | 0.029 | 3.000 | 46.895 | -86.698 |
| Rain | 0.012 | 0.000 | NA | NA | NA | NA | NA | NA | 0.009 | 3.000 | 46.628 | -86.165 |
| Rain | 0.005 | NA | 0.003 | NA | NA | NA | NA | NA | 0.005 | 3.000 | 46.579 | -86.067 |
| Rain | -0.031 | 0.000 | NA | 0.027 | NA | NA | NA | NA | 0.046 | 4.000 | 47.123 | -84.342 |
| Rain | -0.037 | NA | 0.004 | 0.025 | NA | NA | NA | NA | 0.038 | 4.000 | 47.012 | -84.120 |
| Rain | 0.009 | 0.000 | 0.002 | NA | NA | NA | NA | NA | 0.012 | 4.000 | 46.662 | -83.420 |
| Rain | -0.036 | 0.000 | 0.003 | 0.028 | NA | NA | NA | NA | 0.050 | 5.000 | 47.180 | -81.361 |
| Rain | 0.037 | NA | NA | NA | 0.001 | -0.006 | -0.038 | -0.023 | 0.137 | 6.000 | 48.429 | -80.438 |
| Rain | -0.022 | NA | NA | 0.041 | 0.001 | -0.009 | -0.047 | -0.028 | 0.218 | 7.000 | 49.708 | -79.194 |
| Rain | 0.034 | NA | 0.003 | NA | 0.000 | -0.005 | -0.039 | -0.023 | 0.142 | 7.000 | 48.497 | -76.772 |
| Rain | 0.039 | 0.000 | NA | NA | 0.001 | -0.004 | -0.038 | -0.023 | 0.141 | 7.000 | 48.477 | -76.731 |
| Rain | -0.023 | 0.000 | NA | 0.043 | 0.000 | -0.006 | -0.046 | -0.028 | 0.228 | 8.000 | 49.867 | -75.264 |
| Rain | -0.029 | NA | 0.004 | 0.042 | -0.001 | -0.007 | -0.048 | -0.028 | 0.227 | 8.000 | 49.853 | -75.236 |
| Rain | 0.035 | 0.000 | 0.003 | NA | 0.000 | -0.003 | -0.038 | -0.023 | 0.144 | 8.000 | 48.531 | -72.591 |
| Rain | -0.029 | 0.000 | 0.003 | 0.044 | -0.001 | -0.005 | -0.047 | -0.028 | 0.234 | 9.000 | 49.979 | -70.709 |

Species and site traits


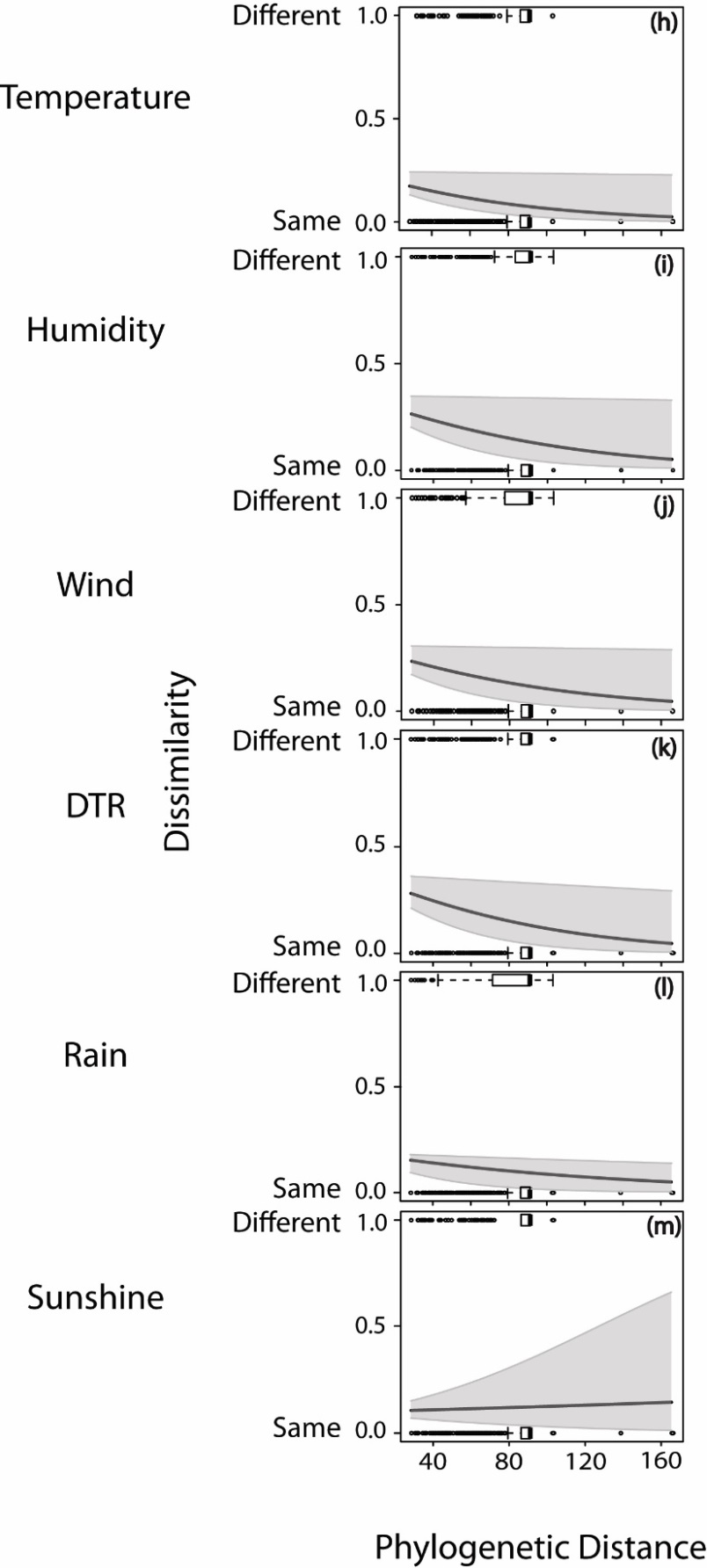


Fig C. The effect of phylogenetic relatedness on whether species are affected by the same climate variables. Displays the pairwise phylogenetic distance in relation to whether species are impacted by the same climate variables (i.e. whether both species are or are not affected by that climate variable, or if one species is affected by that climate variable, but the other is not) with 95% confidence intervals. The boxplots show the distribution of the underlying data.

Table F. Model selection table for all combinations of species characteristics explaining variation in climate vulnerabilities. Shows the best to worst models for each of the six climate variables (ranked by AICc). Each model included either all or none of the habitat characteristics (including open, urban, wet and woodland habitats).The column Int. shows the intercept value, df the degrees of freedom and logLik the log-likelihood value. DTR is the daily temperature range.

| **Int.** | **Body condition** | **Migration** | **Survival** | **Open** | **Urban** | **Wet** | **Wood**  **land** | **R^2^** | **df** | **logLik** | **AICc** |
| --- | --- | --- | --- | --- | --- | --- | --- | --- | --- | --- | --- |
| -0.017 | NA | NA | NA | NA | NA | NA | NA | 0.000 | 2.000 | -22.121 | 48.584 |
| -0.056 | NA | 0.026 | NA | NA | NA | NA | NA | 0.012 | 3.000 | -21.894 | 50.495 |
| -0.013 | 0.000 | NA | NA | NA | NA | NA | NA | 0.001 | 3.000 | -22.100 | 50.907 |
| -0.009 | NA | NA | -0.004 | NA | NA | NA | NA | 0.000 | 3.000 | -22.118 | 50.943 |
| -0.088 | NA | 0.029 | 0.014 | NA | NA | NA | NA | 0.013 | 4.000 | -21.872 | 52.957 |
| -0.067 | 0.000 | 0.030 | NA | NA | NA | NA | NA | 0.013 | 4.000 | -21.877 | 52.967 |
| -0.054 | -0.001 | NA | 0.027 | NA | NA | NA | NA | 0.002 | 4.000 | -22.078 | 53.369 |
| -0.086 | 0.000 | 0.029 | 0.013 | NA | NA | NA | NA | 0.013 | 5.000 | -21.872 | 55.619 |
| 0.014 | NA | NA | NA | -0.460 | -0.022 | -0.030 | -0.012 | 0.031 | 6.000 | -21.524 | 57.758 |
| -0.060 | NA | 0.029 | NA | -0.434 | -0.006 | -0.019 | 0.012 | 0.042 | 7.000 | -21.303 | 60.340 |
| 0.014 | 0.000 | NA | NA | -0.459 | -0.020 | -0.029 | -0.008 | 0.032 | 7.000 | -21.507 | 60.748 |
| 0.014 | NA | NA | 0.000 | -0.460 | -0.022 | -0.030 | -0.012 | 0.031 | 7.000 | -21.524 | 60.781 |
| -0.069 | 0.000 | 0.033 | NA | -0.432 | -0.006 | -0.019 | 0.012 | 0.043 | 8.000 | -21.292 | 63.551 |
| -0.076 | NA | 0.031 | 0.010 | -0.435 | -0.008 | -0.021 | 0.010 | 0.043 | 8.000 | -21.294 | 63.553 |
| -0.041 | -0.001 | NA | 0.041 | -0.467 | -0.027 | -0.035 | -0.017 | 0.034 | 8.000 | -21.462 | 63.890 |
| -0.071 | 0.000 | 0.032 | 0.002 | -0.433 | -0.007 | -0.019 | 0.012 | 0.043 | 9.000 | -21.292 | 67.013 |

Table G. The delta AICc values for the two population-level predictor variables (habitat and density) tested for each climate variable. Here, the null model does not contain any predictors but does include the random effect of species (*Sensitivity ~ 1 + (1|species)*), the habitat model includes the explanatory variable habitat (wet or dry) and includes a random slope term across all species (*Sensitivity ~ Habitat + (1|species) + (0 + Habitat|species)*), null habitat model is the same as the habitat model but does not include a random slopes term (*Sensitivity ~ Habitat + (1|species)*) and the density model includes the explanatory variable with a random intercept term(*Sensitivity ~ Density + (1|species)*). A delta AICc value of 0 indicated that it was the best model.

|  | **Model delta AICc Values** | | | |
| --- | --- | --- | --- | --- |
| **Climatic Variable** | **Null** | **Habitat** | **Null Habitat** | **Density** |
| Humid | 6.9 | 10.6 | 8.5 | 0 |
| Rain | 0.5 | 2.0 | 0 | 2.5 |
| Temperature | 3.8 | 1.7 | 0 | 3.7 |
| Sunshine | 0 | 3.8 | 1.7 | 1.5 |
| Wind | 0 | 1.7 | 0.1 | 2.1 |
| DTR | 0 | 3.7 | 1.6 | 0.4 |
| Vulnerability | 0 | 4.0 | 1.9 | 2.0 |

In populations with higher densities (i.e. better habitats), birds experienced stronger declines in body condition per percent change in humidity compared with those in lower densities (slope of ‑1.03 change in sensitivity per birds/net metre/day; Fig D).


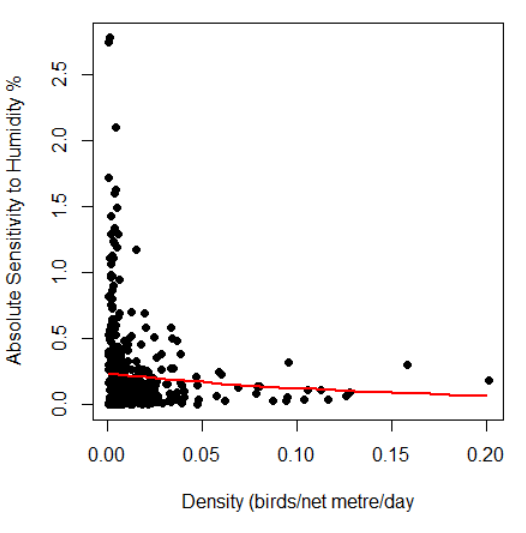


**Fig D.** **The relationship between body condition sensitivity to humidity (as a percent of total boy mass) and habitat density (slope = -1.03).**Nine species were found to have more similar sensitivities at sites that were closer together (Table H). As we did not take into account the effects of multiple testing, some of these species would likely be significant due to chance. However, five species (the common chaffinch, garden warbler, short-toed treecreeper, sedge warbler and willow warbler) all had multiple positive relationships for different climate variables (or the only climate variable they were sensitive to), suggesting that these similarities are not due to chance. Overall vulnerabilities were more similar in populations that were closer together for 24% (7 out of 29) of species (Table H).

Table H. Species with significant relationships between distance (km) and dissimilarity in sites sensitivities and vulnerabilities. Species that have population sensitivities which are more similar at closer distances for each climatic variable are marked with an X. The grey cells indicate that the species was not affected by those climate variables (i.e. they did not have a climate window for that climatic variable).

| **Species** | **Humid** | **Temperature** | **DTR** | **Rain** | **Wind** | **Sunshine** | **Vulnerability** |
| --- | --- | --- | --- | --- | --- | --- | --- |
| Willow warbler |  |  | X | X |  |  | X |
| Common chaffinch | X |  |  | X |  |  |  |
| Common chiffchaff |  | X |  |  |  |  | X |
| Bearded reedling |  | X |  |  |  |  | X |
| Garden warbler |  |  | X |  |  |  | X |
| Short-toed treecreeper | X |  |  |  |  | X |  |
| Sedge warbler |  |  |  | X | X |  |  |
| Eurasian tree sparrow |  |  |  |  | X |  |  |
| European robin |  |  |  |  |  | X |  |
| Eurasian blackcap |  |  |  |  |  |  | X |
| Bluethroat |  |  |  |  |  |  | X |
| Common grasshopper warbler |  |  |  |  |  |  | X |
